# *BrTTG1* regulates seed coat proanthocyanidin formation through a direct interaction with structural gene promoters of flavonoid pathway and glutathione S-transferases in *Brassica rapa* L.

**DOI:** 10.3389/fpls.2024.1372477

**Published:** 2024-04-04

**Authors:** Wenju Zhao, Xiaojuan Li, Junqin Wen, Quanhui Li, Shuanling Bian, Yanjing Ren

**Affiliations:** ^1^ Qinghai University, Academy of Agriculture and Forestry Sciences of Qinghai Province, Laboratory of Research and Utilization of Germplasm Resources in Qinghai-Tibet Plateau, Qinghai, Xining, China; ^2^ Key Laboratory of Germplasm Resources Protection and Genetic Improvement of the Qinghai-Tibet Plateau in Ministry of Agriculture and Rural, Qinghai, Xining, China

**Keywords:** BrTTG1, proanthocyanidins, flavonoid biosynthesis pathway, *Brassica rapa* L., promoters, gene overexpression

## Abstract

**Introduction:**

Seed coat color is a significant agronomic trait in horticultural crops such as *Brassica rapa* which is characterized by brown or yellow seed coat coloration. Previous *Brassica rapa* studies have shown that *BrTTG1* is responsible for seed coat proanthocyanidin formation, which is dependent on the MYB-bHLH-WD40 complex, whereas some studies have reported that TRANSPARENT TESTA GLABRA 1 (TTG1) directly interacts with the structural gene promoters of the flavonoid pathway.

**Methods:**

Herein, the brown-seeded inbred B147 and ttg1 yellow-seeded inbred B80 mutants were used as plant materials for gene expression level analysis, gene promoter clone and transient overexpression.

**Results:**

The analysis identified eleven structural genes involved in the flavonoid biosynthesis pathway, which are potentially responsible for *BrTTG1*- dependent seed coat proanthocyanidin formation. The promoters of these genes were cloned and cis-acting elements were identified. Yeast one-hybrid and dual-luciferase assays confirmed that BrTTG1 directly and independently interacted with *proCHS-Bra008792, proDFR-Bra027457, proTT12-Bra003361, proTT19-Bra008570, proTT19-Bra023602* and *proAHA10-Bra016610*. A TTG1-binding motif (RTWWGTRGM) was also identified. Overexpression of TTG1 in the yellow-seed *B. rapa* inbred induced proanthocyanidin accumulation by increasing the expression levels of related genes.

**Discussion:**

Our study unveiled, for the first time, the direct interaction between TTG1 and the promoters of the flavonoid biosynthesis pathway structural genes and glutathione S-transferases in *Brassica rapa*. Additionally, we have identified a novel TTG1-binding motif, providing a basis for further exploration into the function of TTG1 and the accumulation of proanthocyanidins in seed coats.

## 1 Introduction

Seed coat color is an important economic trait in *Brassica* crops and is closely associated with seed quality in terms of proanthocyanidins (PAs) (Ren et al., 2021), seed oil (Abbadi and Leckband, 2011), seed protein (Li et al., 2012), seed fiber (Wang et al., 2016), phenolic compound, and tannin contents (Zhai et al., 2020; Ding et al., 2021; Xie et al., 2020; Chao et al., 2022). *Brassica rapa* is a major rapeseed and vegetable crop in the Tibetan Plateau and northern China, with a short growth period and resistance to barren fields. *B. rapa* (AA = 20) is considered the original parent species of *Brassica napus* (AACC = 38) and *Brassica juncea* (AABB = 36) (Ren et al., 2021). Thus, the significance of studies on the seed coat color of *B. rapa* extends to other *Brassica* crops.

The pigment deposits responsible for seed coat color are attributed to the accumulation of PAs in the innermost cell layer of the testa (Debeaujon et al., 2000; Dixon et al., 2005; Lepiniec et al., 2006; Zhang et al., 2022). Several studies have shown that seed coat PA formation is directly controlled by structural genes involved in the flavonoid biosynthesis pathway (Akhov et al., 2009; Auger et al., 2010; Li et al., 2010; Lian et al., 2017; Ren et al., 2021; Xie et al., 2020). Early biosynthetic genes (EBGs) produce common precursors of PAs, and late biosynthetic genes (LBGs) are regulated by the ternary MYB-bHLH-WD40 complex (Xu et al., 2014). PA precursors are synthesized on the cytosolic surface of the endoplasmic reticulum and transported to the vacuole via *glutathione S-transferases* (*GSTs*) and membrane transporters regulated by genes such as including *TT12*, *TT19*, and *AHA10* (Zhao, 2015).

TRANSPARENT TESTA GLABRA 1 (TTG1), a WD40 repeat protein involved in MYB-bHLH-WD40 (MBW) complexes, can regulate both the specific activity (i.e., interactions with other proteins or DNA) and quantity (e.g., stability and localization) of MBW complexes (Baudry et al., 2004; Koes et al., 2005; Thévenin et al., 2012; Xu et al., 2015) while being essential for preventing the effects of plant-specific negative regulators (Xu et al., 2015). Gonzalez et al. (2009) reported that the particular epidermal cell fate is specified by TTG1 in conjunction with a specific MYB and bHLH class transcription factor complex. Jia et al. (2021) showed that the tissue-specific pattern of PA biosynthesis relies more on R2R3-MYB factors, whereas TTG1 is an indispensable and constant regulator of MBW complexes.

Contrary to Ke et al. (2023) who showed that WD40 proteins lack transcriptional regulatory ability, we predicted and identified six TTG1-dependent structural genes involved in the flavonoid biosynthesis pathway. Yeast one-hybrid (Y1H) and dual-luciferase assays confirmed that TTG1 directly interacts with *proCHS-Bra008792*, *proDFR-Bra027457*, *proTT12-Bra003361*, *proTT19-Bra008570*, *proTT19-Bra023602*, and *proAHA10-Bra016610* and induces their expression. Our study provides insights on the function of TTG1 and the PAs’ regulatory impact on seed coat color research.

## 2 Materials and methods

### 2.1 Plant materials and growth conditions

Brown-seeded inbred B147 and *ttg1* yellow-seeded inbred B80 mutants were used as plant materials, and their seeds were sown in a greenhouse in the winter of 2016 for vernalization. Seedlings were subsequently transplanted and transferred to a plastic shed in the spring of 2017 for artificial pollination during blossom in Yangling, Shaanxi province, China. Seeds of different development stages were collected at 10, 14, 18, 22, 26, 30, and 34 days after flowering (DAF), frozen in liquid nitrogen, and immediately stored in a −80°C freezer (Sanyo, Japan). Three biological replicates were analyzed for each sample.

Leaves from tobacco (*Nicotiana benthamiana*) and yellow-seeded inbred B80 *B. rapa ttg1* mutant plants were used for transient expression. Tobacco and *B. rapa* seeds were sown separately in the culture soil after soaking for 24 h. Fifteen-day-old tobacco seedlings were transplanted into plastic bowls (10 cm ×10 cm) and placed in the culture room under a 16-h light/8-h dark photoperiod, 22°C temperature and 60% humidity. Fourteen-day-old *B. rapa* seedlings were used for the transient expression analysis.

### 2.2 DNA, RNA extraction, and gene expression

Genomic DNA was extracted from fresh young leaves using a modified version of the cetyltrimethylammonium bromide method (Porebski et al., 1997). Total RNA extraction and cDNA synthesis were performed as previously described (Ren et al., 2017). Gene expression analysis was performed using quantitative real-time PCR (RT-qPCR), and a housekeeping gene encoding glyceraldehyde-3-phosphate dehydrogenase (GAPDH; GO0048316) was used as a reference gene (Li et al., 2016). The RT-qPCR reaction was performed in 20-µL volume in a Roche LightCycler 480 Real-Time PCR Detection System with three replications as previously described (Ren et al., 2017). Relative expression level of genes was calculated using the 2^−ΔΔCt^ method (Livak and Schmittgen, 2001). The specificity of primers (**Supplementary Table 1**) used for the amplifications was confirmed by melting curve analysis.

### 2.3 Cloning and analysis of gene promoters

Based on the location of the ATG initiation codon of each gene in the BRAD database, 1,500- to 2,000-bp sequences upstream the ATG were screened to design promoter cloning primer (**Supplementary Table 2**) using Primer Premier 5.0. PCR amplification procedure was performed in a 25-µL volume according to previously described instructions. A tail A was added to the purified PCR amplification products, which were subsequently cloned to the pMD19-T simple vector and transformed to *Escherichia coli* strain DH5α for sequencing. Promoter function was predicted using PlantCARE database online tools. Visualization of the cis-acting elements was performed using the online software Gene Structure Display Server.

### 2.4 Yeast one-hybrid assays

For the Y1H assays, promoters were inserted into the pAbAi vector, and, then, pAbAi-promoter recombination plasmids were transformed into the Y1H Gold yeast strain for aureobasidin A (AbA; Clontech, USA) concentration screening. Confirmed transformants were spread on SD/-Ura, SD/-Ura/AbA (150 ng/mL), and SD/-Ura/AbA (300 ng/mL) culture media, and the optimal AbA concentration was determined on the basis of colony growth.

The *TTG1*-ORF was integrated into the pGADT7 vector, and the pGADT7-TTG1 recombinant plasmid was transformed into a recombinant yeast strain containing pAbAi promoters. Recombinant transformants were spread on SD/-Leu/AbA culture medium, whereas SD/-Leu without AbA was used as a control. The interaction between BrTTG1 and its promoter was assessed on the basis of the colony growth. The combination of pGADT7-p53 + pAbAi-*p53* was used as a positive control, and the combination of empty pGADT7 + pAbAi-promoters was used as a negative control. All primers used for recombinant plasmid construction in the Y1H assay are listed in **Supplementary Table 3**.

### 2.5 Dual-luciferase assays

For the dual-luciferase assays, *BrTTG1*-ORF and promoters were introduced into pGreenII 62-SK and pGreenII 0800-LUC vectors by homologous recombination. The recombinant plasmids were then separately transformed into GV3101. pGreenII 62-SK-*BrTTG1* was used as an effector, and promoter-pGreenII 0800-LUC was used as a reporter. All primers used for the construction of recombinant plasmids in the dual-luciferase assay are listed in **Supplementary Table 4**.

Six independent tobacco (*Nicotiana benthamiana*) leaves were transfected during the transient expression assays. The transfected plants were kept in the dark overnight and then transferred to the culture room for 2 days. Luciferase activity was evaluated using a TransDetect Double-Luciferase Reporter Assay Kit (Promega, USA), and the regulatory relationship between TTG1 and the promoters was determined on the basis of the ratio of firefly luciferase activity to sea Renilla luciferase activity.

### 2.6 Promoters activity assays

Based on the distribution of cis-acting elements in the promoter sequences, several fragments of different lengths containing *Hind*III and *SaI*I restriction sites were cloned and introduced into the pcambia1391-GUS vector by homologous recombination individually. All primers used for recombinant plasmid construction in promoter activity assays are listed in **Supplementary Table 5**.

Transient expression was conducted in tobacco leaves, and six independent leaves were transfected as described above. The *promoter: GUS-*transfected leaves were dyed by soaking in β-glucuronidase (GUS) staining buffer [0.05% m/v 5-bromo-4-chloro-3-indolyl-β-glucuronide, 100 mM NaH_2_PO_4_·H_2_O, 10 mM EDTA, 0.5 mM K_4_FE(CN)_6_·3H_2_O, 0.1% Triton] and vacuum penetration for 30 min followed by staining at 37°C for 24 h. The stained leaves were soaked in 70% alcohol several times until they were completely decolorized, and photographs were taken. The reaction of GUS with 4-methylumbelliferyl β-D-glucuronide (MUG) resulted in the production of the fluorescent substance 4-methylumbelliferone (MU), and its content was determined by a fluorospectrophotometer. Thus, following the method described by Jefferson et al. (1987), the GUS content was quantified on the basis of the MU content of the plant total protein per minute. The protein content was determined using the Bradford method (Bradford, 1976). Our strategy followed the ones reported by Debeaujon et al. (2003); Thévenin et al. (2012), and Xu et al. (2014) with minor modifications. TTG1-binding motif enrichment was predicted using the MEME-ChiP (Bailey and Machanick, 2012).

### 2.7 Transient overexpression assays in *B. rapa*

The CDS of *TTG1* was inserted into the pVBG2307 vector by homologous recombination, and the recombinant plasmid pVBG2307-*TTG1* was transformed into GV3101 for transient injection into 14-day-old *B. rapa* cotyledons. GV3101 cells with an optical density (OD_600_) set to approximately 0.600 using a UV spectrophotometer (Thermo Fisher Scientific, USA) were injected into the leaves. Transformed plants were kept in the dark for 12 h, and, then, the light conditions were changed to 16-h light/8-h dark photoperiod for 2 days. Three individually transformed cotyledons and their corresponding leaves were collected for gene expression analysis and PA content determination.

### 2.8 Total proanthocyanidin analysis

Total PA extraction and content analysis were performed using the Plant PA Content Detection kit (Solarbio, China, BC1355). A quantity of 100 mg of dried transformed leaves was collected and dissolved in 1 mL of extract buffer, and, then, the ultrasonic method (300 W, 25°C, 30 min) was used for extraction followed by a centrifugation at 12,000 rpm at 25°C for 10 min. The supernatant was collected and diluted in 1 mL of extraction buffer, and the absorbance was measured at 500 nm.

### 2.9 Data statistics

All data calculations were performed using Microsoft Excel 2019, and column diagrams were drawn using Origin 2021. Significance analysis was performed using SPSS version 20.0.

## 3 Results

### 3.1 Prediction of structural genes involved in the BrTTG1-dependent flavonoid biosynthesis pathway

**Figure 1:**
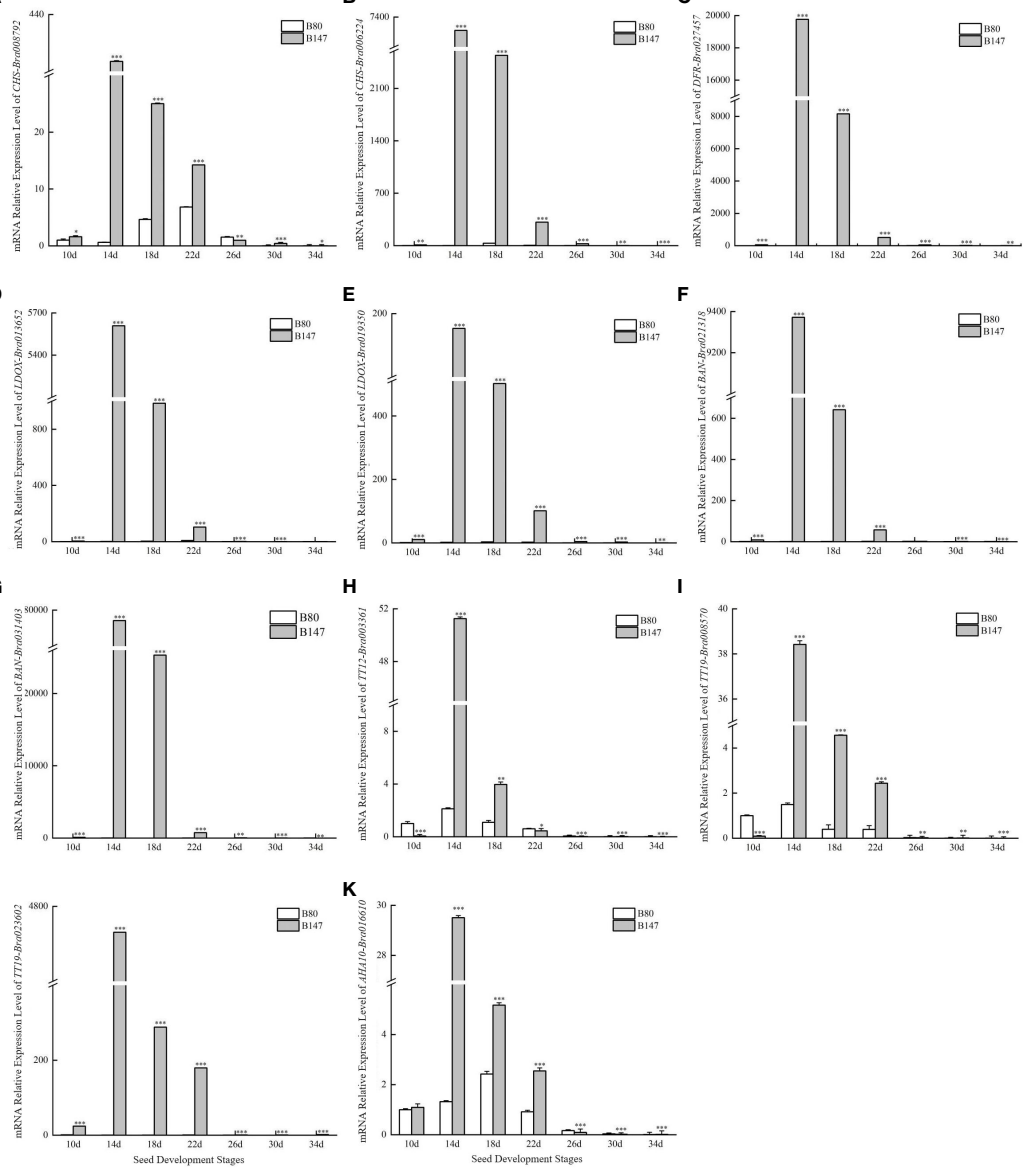
Expression levels of 11 structural genes involved in the flavonoid biosynthesis pathway dependent on TTG1 at seven different development stages seeds. **(A)** *CHS-Bra008792*; **(B)** *CHS-Bra006224*; **(C)** *DFR-Bra027457*; **(D)** *LDOX*-*Bra013652*; **(E)** *LDOX*-*Bra019350*; **(F)** *BAN*-*Bra021318*; **(G)** *BAN*-*Bra031403*; **(H)** *TT12-Bra003361*; **(I)** *TT19-Bra008570*; **(J)** *TT19-Bra023602*; **(K)** *AHA10-Bra016610*. ***represents significantly difference when p-value was 0.005.

Seeds from brown-seeded inbred B147 and yellow-seeded inbred B80 plants collected at seven different developmental stages were used to predict the structural genes involved in the TTG1-dependent flavonoid biosynthesis pathway. Expression level analysis showed that 11 of the 22 structural genes had significant higher expression levels in B147 than in B80 at seven different developmental stages (10, 14, 18, 22, 26, 30, and 34 days after flowering) (**Figure 1**, **Supplementary Figure 1**). These were *CHS-Bra008792*, *CHS-Bra006224*, *DFR*-*Bra027457*, *LDOX*-*Bra013652*, *LDOX*-*Bra019350*, *BAN*-*Bra021318*, *BAN*-*Bra031403*, *TT12-Bra003361*, *TT19-Bra008570*, *TT19-Bra023602*, and *AHA10-Bra016610*. We speculated that these TTG1-dependent genes probably regulate seed coat PA formation in *B. rapa*.

### 3.2 Analysis of candidate target genes promoters

**Figure 2:**
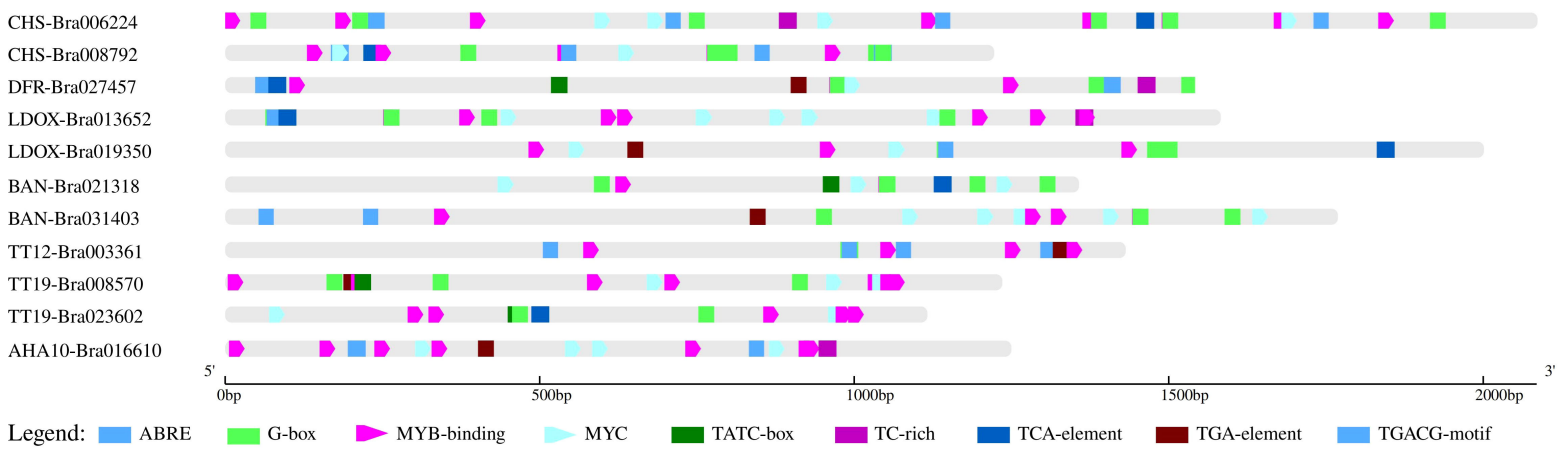
Cis-acting element analysis of 11 candidate target genes promoters.

Based on the reference *B. rapa* Chiifu genome from the BRAD database, a total of 11 candidate target gene promoters of brown-seeded B147 plants were cloned and analyzed. These were pro*CHS*-*Bra008792*, pro*CHS*-*Bra006224*, pro*DFR*-*Bra027457*, pro*LDOX*-*Bra013652*, pro*LDOX*-*Bra019350*, pro*BAN*-*Bra021318*, pro*BAN*-*Bra031403*, pro*TT12*-*Bra003361*, pro*TT19*-*Bra008570*, pro*TT19*-*Bra023602*, and pro*AHA10*-*Bra016610* and had a length of 1,222 bp, 2,085 bp, 1,582 bp, 2,000 bp, 1,357 bp, 1,768 bp, 1,431 bp, 1,235 bp, 1,116 bp, and 1,249 bp, respectively. All promoter sequences are provided as **Supplementary Materials**. Analysis of these 11 promoter sequences revealed 98, 131, 77, 123, 140, 71, 102, 127, 100, 77, and 76 cis-acting elements, respectively. These elements are implicated in light response, circadian regulation, jasmonic acid response, salicylic acid response, regulation of zeolin metabolism, abscisic acid response, auxin-response, defense and stress responses, and anaerobic induction and constitute core elements of transcription initiation, promoter, and enhancer regions (**Figure 2**).

Furthermore, considering that PA formation is regulated by the MYB and bHLH/MYC transcription factors, we analyzed their binding sites separately. Results revealed no MYC-binding sites in *proTT12*-Bra003361, whereas the remaining 10 promoters contained both MYB and MYC-binding sites.

### 3.3 Interaction analysis of TTG1 with the candidate target gene promoters

**Figure 3:**
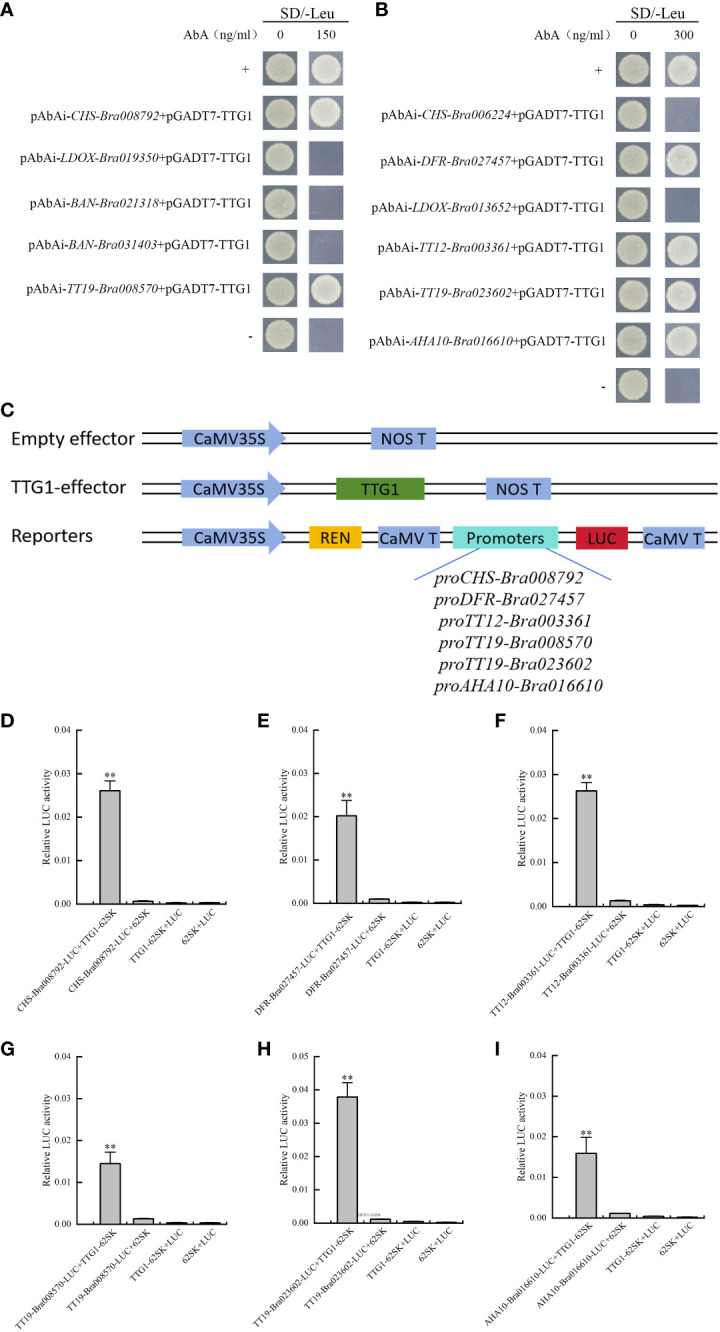
TTG1 directly regulates structural genes promoters of the flavonoid biosynthesis pathway and glutathione S-transferases. **(A, B)** TTG1 binding with *proCHS-Bra008792*, *proDFR-Bra027457*, *proTT12-Bra003361*, *proTT19-Bra008570*, *proTT19-Bra023602*, and *proAHA10-Bra016610*, separately. **(C)** Schematic diagrams of the effector and reporter plasmids used for the dual-luciferase assay. REN represents Renilla luciferase. LUC represents firefly luciferase. **(D–I)** TTG1 activates *proCHS-Bra008792*, *proDFR-Bra027457*, *proTT12-Bra003361*, *proTT19-Bra008570*, *proTT19-Bra023602*, and *proAHA10-Bra016610* separately in dual-luciferase assay. The empty effector, empty reporters and empty effector with empty reporter were used as controls, separately. ** represents significantly difference when p-value was 0.01.

Eleven pAbAi promoter recombinant plasmid autoactivation assays were performed. Five recombinant yeast strains (pAbAi-*CHS-Bra008792*, pAbAi-*BAN-Bra021318*, pAbAi-*BAN-Bra031403*, pAbAi-*LDOX-Bra019350*, and pAbAi-*TT19-Bra008570*) failed to grow in the SD/-Ura/AbA (150 ng mL^−1^) culture medium. Six recombinant yeast strains, including pAbAi-*CHS-Bra006224*, pAbAi-*DFR*-*Bra027457*, pAbAi-LDOX-Bra013652, pAbAi-*TT12-Bra003361*, pAbAi-*TT19-Bra023602*, and pAbAi-*AHA10-Bra016610*, failed to grow in the SD/-Ura/AbA (300 ng mL^−1^) culture medium (**Supplementary Figure 2**). Based on the autoactivation activity assay results, the interaction of TTG1 with the candidate target gene promoters was assessed using Y1H assays in two types of culture media. The results showed that TTG1 bound to *proCHS-Bra008792*, *proDFR-Bra027457*, *proTT12-Bra003361*, *proTT19-Bra008570*, *proTT19-Bra023602*, and *proAHA10-Bra016610* (**Figures 3A, B**). A dual-luciferase assay was performed to confirm that TTG1 directly interacts with the above six promoters and induces their expression (**Figures 3C-I**).

### 3.4 Analysis of the minimal active fragment of the promoter

**Figure 4:**
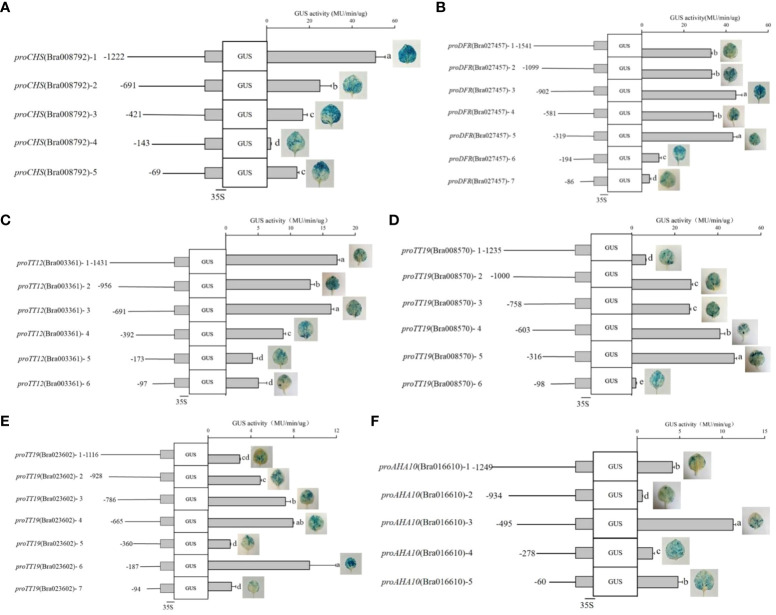
GUS staining and activity of the target gene promoters. **(A)** *proCHS-Bra008792*; **(B)** *proDFR-Bra027457*; **(C)** *proTT12-Bra003361*; **(D)** *proTT19-Bra008570*; **(E)** *proTT19-Bra023602*; **(F)** *proAHA10-Bra016610.* Left panels: The different promoters issued from the 5′-end deletion series were fused to the 35S cauliflower mosaic virus minimal promoter upstream of the GUS reporter gene. The transient expression assays was performed in tobacco leaves. Right panels: Column diagram represent GUS activity in transformed tobacco leaves driven by the different promoters fragment. Lowercase letters indicate significant difference.

To characterize the DNA regions involved in the regulation of *proCHS-Bra008792*, *proDFR-Bra027457*, *proTT12-Bra003361*, *proTT19-Bra008570*, *proTT19-Bra023602*, and *proAHA10-Bra016610* activity by TTG1, transient transformation was performed in tobacco leaves. Based on the cis-acting element location in the promoter sequences, several fragments of different lengths were cloned into the pcambia1391-GUS vector for transient transformation, using GUS as a reporter. The key deletion fragments were characterized on the basis of GUS staining and the drastic changes in activity. GUS activity kept decreasing from *proCHS-Bra008792-1* (−1,222 bp) to *proCHS-Bra008792-4* (−143 bp) with the blue color of GUS staining gradually becoming lighter. Removal of an additional 278-bp fragment (*proCHS-Bra008792-4*) led to a strong decrease (~88.7%) in activity. These results indicate that this fragment contains the minimal information necessary to drive transcriptional activity. Intriguingly, *proCHS-Bra008792-5* led to a stronger increase (~ 6.43-fold) compared to the GUS activity of *proCHS-Bra008792-4*, suggesting that some regions upstream of *proCHS-Bra008792-5* have a negative impact on *proCHS-Bra008792* activity (**Figure 4A**). Among the seven fragments of *proDFR-Bra027457*, GUS activity was higher than that of *proDFR-Bra027457-1* (−1,541 bp) and *proDFR-Bra027457-5* (−319 bp) and then decreased in *proDFR-Bra027457-6* (−194 bp). Removal of an additional fragment of 125 bp (*proDFR-Bra027457-6*) led to a strong decrease (~81.2%) in the activity (**Figure 4B**), suggesting that this fragment contains the minimal information necessary to drive transcriptional activity. Analysis of six fragments of *proTT12-Bra003361* led to the identification of a 219-bp promoter fragment from *proTT12-Bra003361-4* (−392 bp) to *proTT12-Bra003361-5* (−173 bp) (**Figure 4C**). Analysis of the *proTT19-Bra008570* identified a 218-bp promoter fragment from *proTT19-Bra008570-5* (−316 bp) to *proTT19-Bra008570-6* (−98 bp), which led to a strong decrease (~95.9%) in activity (**Figure 4D**). Analysis of *proTT19-Bra023602* identified a 219-bp promoter fragment from *proTT19-Bra023602-4* (−665 bp) to *proTT19-Bra023602-5* (−360 bp), which led to a strong decrease (~73.8%) in activity. The removal of an additional fragment of 173 bp of *proTT19-Bra023602-5* led to a strong increase (~3.57-fold) in the GUS activity, suggesting that some regions upstream of *proTT19-Bra023602-6* have a negative impact on *proTT19-Bra023602-5* activity (**Figure 4E**). Five fragments were analyzed in *proAHA10-Bra016610*, and a 217-bp fragment deletion from *proAHA10-Bra016610-3* (−495 bp) to *proAHA10-Bra016610-4* led to a GUS activity decrease, whereas a 218-bp fragment deletion from *proAHA10-Bra016610-4* (−316 bp) to *proAHA10-Bra016610-5* led to a GUS activity increase (**Figure 4F**), indicating that the 218-bp fragment upstream of *proAHA10-Bra016610-5* could possibly have an opposite effect with the 217-bp fragment upstream of *proAHA10-Bra016610-4* in terms of driving the transcriptional activity.

**Figure 5:**
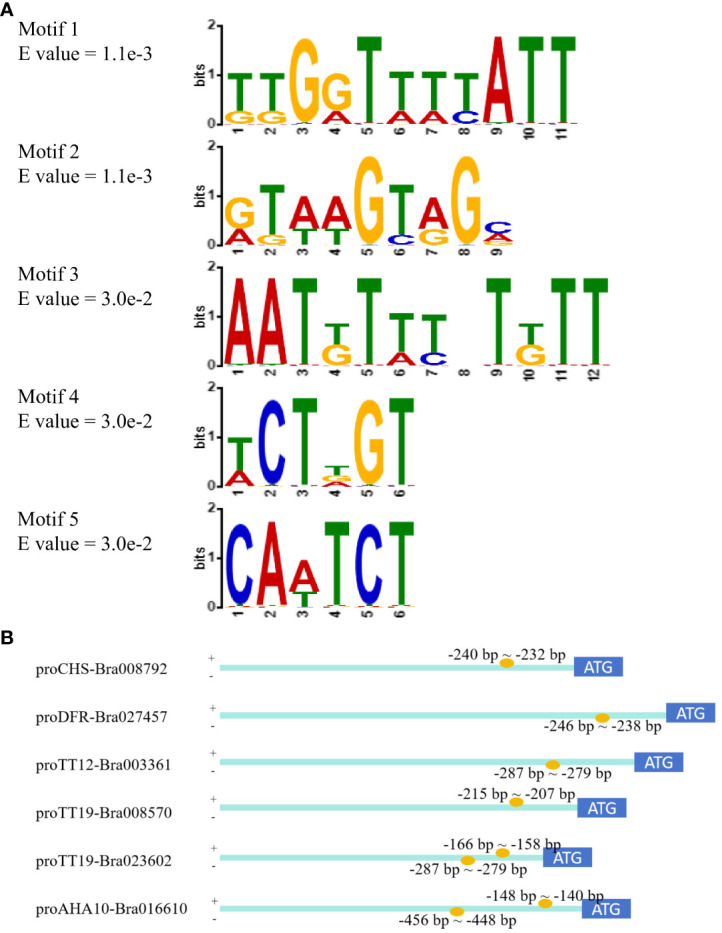
TTG1-binding motifs enrichment analysis by MEME-ChiP. **(A)** Five enriched motifs. **(B)** Positions of motif 2 in promoters.

Based on the GUS activity that was more than 5.0 MU/min/ug, motif enrichment analysis of promoter fragments was performed using MEME-ChiP to determine the sequence of TTG1-binding motifs. Five motifs were significantly enriched, including three novel motifs, a DOF-binding motif, and an NAC-binding motif (**Figure 5A**). Among these motifs, motif 2 (RTWWGTRGM) occurred one or two times per promoter; thus, novel motif 2 was considered the candidate TTG1-binding motif. The position of motif 2 in the promoters was analyzed and is shown in **Figure 5B**. Based on the combined results of GUS staining and GUS activity, fragments containing motif 2 exhibited higher GUS activity in *proCHS-Bra008792-3* (−240 bp ~ −232 bp), *proDFR-Bra027457-5* (−246 bp ~ −238 bp), *proTT12-Bra003361-4* (−287 bp ~ −279 bp), and *proTT19-Bra008570-5* (−215 bp ~ −207 bp). Interestingly, two motif 2 were detected in *proTT19-Bra023602-5* (−287 bp to −279 bp and −166 bp to −158 bp) and *proAHA10-Bra016610-3* (−456 bp to −448 bp and −148 bp to −140 bp), whose GUS activity differed. The *proTT19-Bra023602-5* (−360 bp) containing two motif 2 showed lower GUS activity (2.07 ± 0.05 MU/min/ug), whereas *proTT19-Bra023602-6* (−187 bp) containing one motif 2 showed higher GUS activity (9.48 ± 2.46 MU/min/ug). The *proAHA10-Bra016610-3* (−495 bp) containing two motif 2 showed higher GUS activity (11.29 ± 0.32 MU/min/ug), whereas *proAHA10-Bra016610-4* (-278 bp) containing one motif 2 showed lower GUS activity (1.82 ± 0.23 MU/min/ug).

### 3.5 Overexpression of *BrTTG1* enhanced proanthocyanidin formation

**Figure 6:**
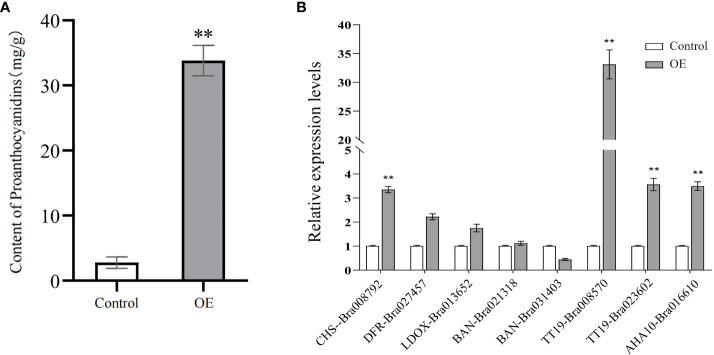
Overexpression of BrTTG1 enhanced proanthocyanidin formation. **(A)** The proanthocyanidin content in transformed seedling cotyledons. **(B)** Expression levels of eight genes involved in flavonoid biosynthesis pathway in transformed seedling cotyledons. ** represents significantly difference when p-value was 0.01.

To clarify the effect of *BrTTG1* on *B. rapa* seed coat color and PA formation, a *BrTTG1* overexpression assay in *B.* rapa seedling cotyledons was carried out. The PAs in untransformed cotyledons were 2.78 ± 0.88 mg/g DW, whereas PAs in transformed cotyledons were 33.80 ± 3.30 mg/g DW. The PAs in the transformed seedling cotyledons were significantly higher than those in the control (**Figure 6A**). In addition, the expression levels of eight genes involved in the flavonoid biosynthesis pathway and Glutathione S-transferases were determined using qRT-PCR. Five target genes including *CHS-Bra008792*, *DFR-Bra027457*, *TT19-Bra008570*, *TT19-Bra023602*, and *AHA10-Bra016610* exhibited a significantly increased expression than that in control, whereas *LDOX-Bra13652*, *BAN-Bra021318*, and *BAN-Bra031403* exhibited no significant changes (**Figure 6B**).

## 4 Discussion

TTG1 was first reported almost half a century ago (Koornneef and Van der Veen, 1978) and has been demonstrated to be involved in the regulation of trichome initiation, seed coat mucilage biosynthesis (Koornneef, 1981), root hair formation (Galway et al., 1994; Zhang et al., 2009), seed development, and PA biosynthesis (Western et al., 2001; Debeaujon et al., 2003; Zhang et al., 2009; Chen et al., 2015; Ren et al., 2017; Yuan et al., 2019). PAs, the end products of the flavonoid biosynthetic pathway, are deposited in the innermost cell layer of the testa giving a characteristic seed coat pigment. Xu et al. (2014) quantified the mRNA accumulation levels of 12 characterized flavonoid biosynthetic genes, leading to PA accumulation between WT and *ttg1* mutants in *Arabidopsis* and found significant decreases in *F3H*, *DFR*, *LDOX*, *BAN*, *TT12*, *TT19*, and *AHA10* mRNA accumulation in *ttg1* mutants compared with the WT, whereas the mRNA accumulation levels of *CHS*, *CHI*, and *TT15* increased in *ttg1* mutants. Ren et al. (2021) showed that the expression levels of EBGs in the flavonoid biosynthetic pathway were downregulated, whereas LBGs were hardly or not expressed at all in *ttg1* mutant seeds at 10, 14, and 28 days after flowering in *Brassica rapa*. Here, seeds at seven different developmental stages were selected to analyze the mRNA accumulation levels of 22 characterized flavonoid biosynthetic genes in *B. rapa*, and 11 genes showed significantly higher expression levels in B147 than in B80 at seven different developmental stages. These results indicate that different copies of the same gene may perform different functions.

During PA formation, TTG1 interacts with R2R3 MYB transcription factors [TRANSPARENT TESTA 2 (TT2), MYB5, MYBPA1, and MYBPA2] and bHLH transcription factors (TT8, GL3, and EGL3) to regulate the expression of LBGs in the flavonoid biosynthesis pathway (Nesi et al., 2000; Baudry et al., 2004; Xu et al., 2014, 2015; Li et al., 2020; Tian and Wang, 2020; Rajput et al., 2022). Anthocyanin biosynthesis and PA biosynthesis possess the same EBGs and LBGs. Jia et al. (2021) showed that light-induced *DcTTG1* regulates anthocyanin biosynthesis in *Dendrobium candidum* by binding to the promoters of *DcCHS2*, *DcCHI*, *DcF3H*, and *DcF3′H* and no direct binding of TTG1 to late anthocyanin biosynthetic gene promoters was observed. In this study, we found that *BrTTG1* directly binds to the promoters of the flavonoid biosynthesis pathway and glutathione S-transferases, including *CHS-Bra008792*, *DFR-Bra027457*, *TT12-Bra003361*, *TT19-Bra008570*, *TT19-Bra023602*, and *AHA10-Bra016610* and regulates PA formation in the presence of MYB and bHLH. Ke et al. (2023) showed that WD40 proteins stabilize the interaction between MYB and bHLH TFs but lack transcriptional regulatory ability. We showed that *BrTTG1* directly bound to the promoters of the flavonoid biosynthesis pathway and promoted PA formation.

During the analysis of the minimally active fragments of promoters, changes in GUS activity did not show an obvious pattern as the promoter fragment decreased, which was similar to the results of the MBW target gene promoter activity analysis reported by Xu et al. (2014). After TTG1-binding motif enrichment, fragments with two motif 2 in *proTT19-Bra023602* and *proAHA10-Bra016610* showed different GUS activities. We subsequently searched for cis-elements in the fragments with two motif 2 in *proTT19-Bra023602* and *proAHA10-Bra016610.* The results showed that MYB-binding sites and bHLH recognition sites were found in *proTT19-Bra023602-6* and *proAHA10-Bra016610-3* fragments with higher GUS activity. No MYB-binding sites or bHLH recognition sites were found in the lower GUS activity fragments of *proAHA10-Bra016610-4*. This finding suggests that the GUS activity of these promoters may be affected by the presence or absence of MYB and bHLH or by other unknown elements.

Our findings indicate that BrTTG1 regulates seed coat PA formation through a direct interaction with structural gene promoters of the flavonoid biosynthesis pathway and glutathione S-transferases.

## 5 Conclusion

*BrTTG1* directly interacts with structural gene promoters of the flavonoid pathway and glutathione S-transferases to regulate seed coat PA formation in *B. rapa*. A TTG1-binding motif (RTWWGTRGM) was identified. Overexpression of *BrTTG1* in yellow seed *B. rapa* inbred plants induced PA accumulation by increasing the expression levels of target genes. Our study revealed, for the first time, a direct interaction between TTG1 and structural gene promoters of the flavonoid biosynthesis pathway and glutathione S-transferases in *B. rapa* and predicted a novel TTG1-binding motif. The above findings provide insights and could be the basis for future studies aiming to study the TTG1 function and PA accumulation in seed coats.

## Data availability statement

The original contributions presented in the study are included in the article/**Supplementary Material**. Further inquiries can be directed to the corresponding author.

## Author contributions

WZ: Conceptualization, Methodology, Software, Validation, Visualization, Writing – original draft. XL: Conceptualization, Methodology, Software, Writing – original draft. JW: Conceptualization, Writing – review & editing. QL: Writing – review & editing. SB: Methodology, Visualization, Writing – review & editing. YR: Conceptualization, Funding acquisition, Methodology, Resources, Supervision, Writing – original draft, Writing – review & editing.

## Supplementary material

Supplementary Figure 1 Expression levels of eleven structural genes involved in the flavonoid biosynthesis pathway independent on TTG1 at seven different development stages seeds. **(A)** *CHS-Bra023441;* **(B)** *CHI-Bra007142*; **(C)** *CHI-Bra007145*; **(D)** *CHI-Bra003209*; **(E)** *F3H-Bra036828*; **(F)** *F3’H-Bra009312*; **(G)** *TT10-Bra037510*; **(H)** *TT15-Bra003021*; **(I)** *TT15-Bra035004*; **(J)** *TT15-Bra038445*; **(K)** *TT15-Bra023594*.

Supplementary Figure 2 AbA concentration screening of eleven pAbAi-promoters recombination plasmids.
