# Supplementary figures and images for: BrTTG1 regulates seed coat proanthocyanidin formation through a direct interaction with structural gene promoters of flavonoid pathway and glutathione S-transferases in Brassica rapa L
Source: Front Plant Sci. 2024 Apr 4;15:1372477. doi: 10.3389/fpls.2024.1372477 (PMC11024264; doi:10.3389/fpls.2024.1372477)

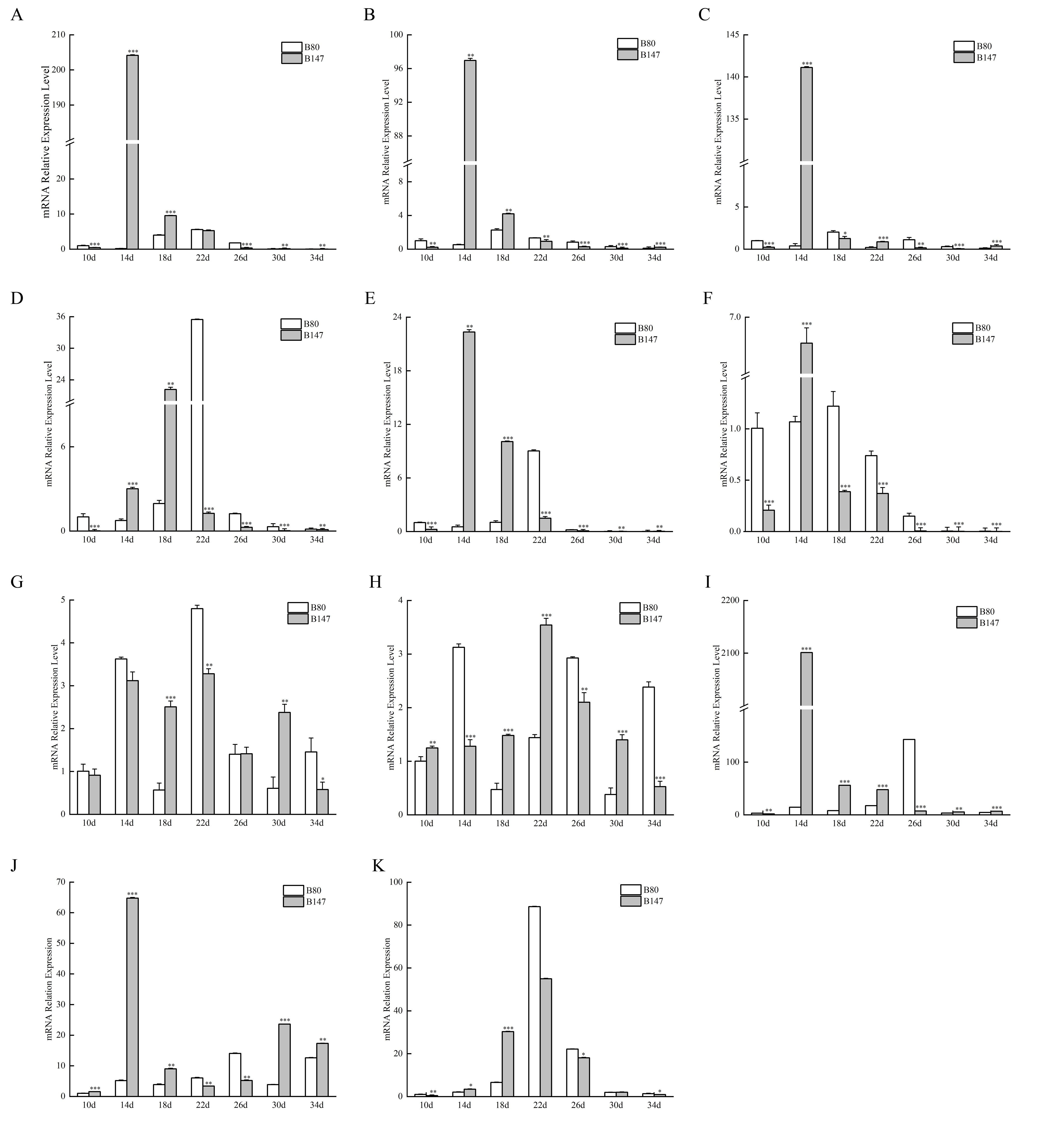

Supplement: Supplementary Figure 1 — Expression levels of eleven structural genes involved in the flavonoid biosynthesis pathway independent on TTG1 at seven different development stages seeds. (A) CHS-Bra023441; (B) CHI-Bra007142; (C) CHI-Bra007145; (D) CHI-Bra003209; (E) F3H-Bra036828; (F) F3’H-Bra009312; (G) TT10-Bra037510; (H) TT15-Bra003021; (I) TT15-Bra035004; (J) TT15-Bra038445; (K) TT15-Bra023594. [file Image_1.jpeg]

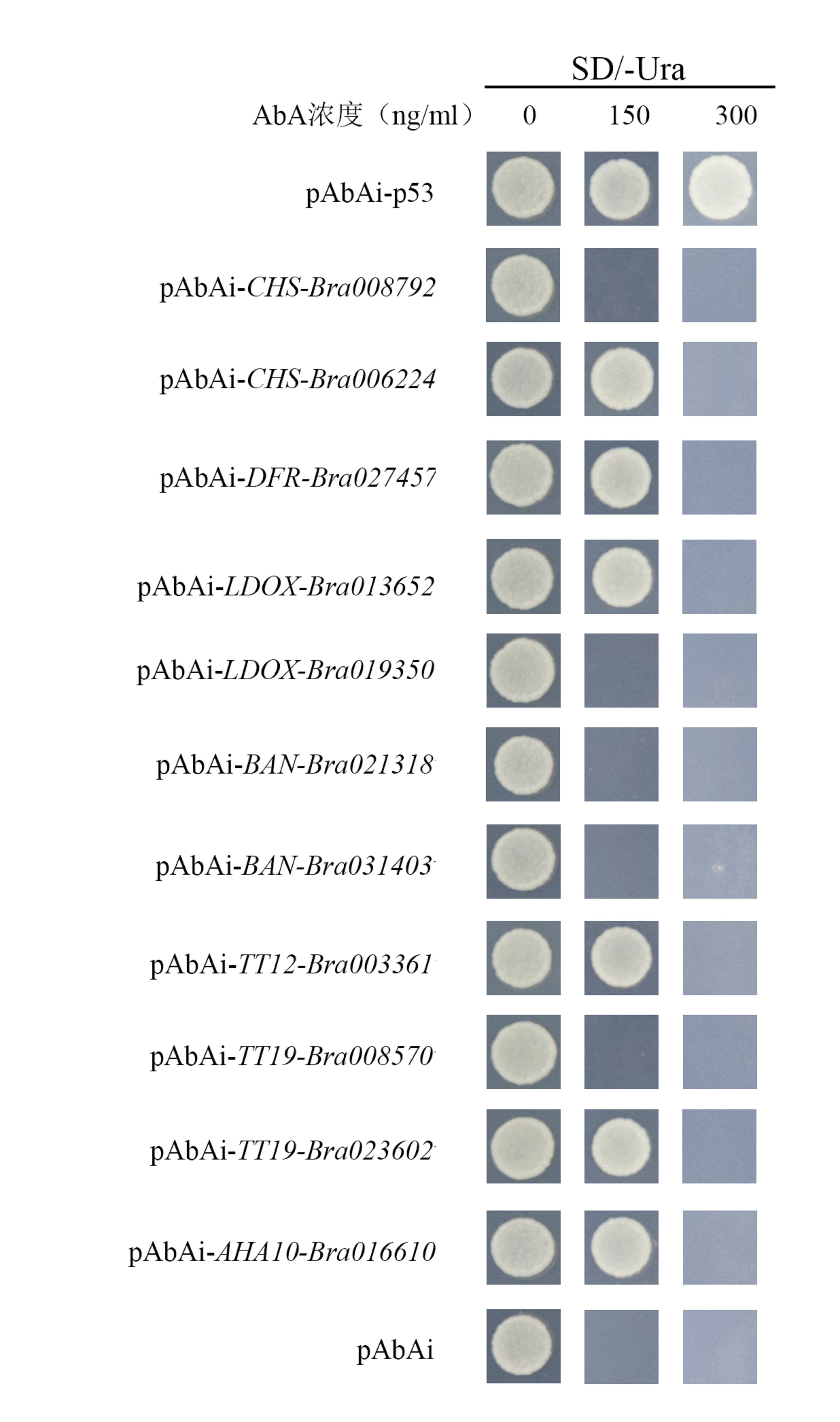

Supplement: Supplementary Figure 2 — AbA concentration screening of eleven pAbAi-promoters recombination plasmids. [file Image_2.tif]
