# Supplementary material for: BrTTG1 regulates seed coat proanthocyanidin formation through a direct interaction with structural gene promoters of flavonoid pathway and glutathione S-transferases in Brassica rapa L
Source: Front Plant Sci. 2024 Apr 4;15:1372477. doi: 10.3389/fpls.2024.1372477 (PMC11024264; doi:10.3389/fpls.2024.1372477)
Supplement: Supplementary file 3 [file Table_1.docx]

Table S1: The gene-specific primers for gene expression analysis used in this study.

| Primer name | Gene ID | Forwarding sequences5’-3’ | Reversed sequence5’-3’ |
| --- | --- | --- | --- |
| *CHS* | Bra008792 | CATCTGACACCCACCTTGACT | ATCTTCTCCGCCTTGAGCCCT |
|  | Bra023441 | ACTTCCGCATCACCAACAG | AGGGACTTCAACCACCACG |
|  | Bra006224 | CCAGACTACTACTTCCGCATC | TTAGGGACTTCAACAACCACA |
| *CHI* | Bra007142 | TGCTTCCATCCTCTTCGCT | GCTAATCTCTCGGCAACACTC |
|  | Bra007145 | ACGACAGAGGAGTTAGCGGAA | GAGAAAGCGAAGAGGATGGAA |
|  | Bra003209 | TTCCTCCAATCCCCTCTTC | TGGTTTTGCCTTTCCACTT |
| *F3H* | Bra036828 | CTTCTTCGCCTTACCTCCT | CTCCTCCGTCACTTTCACC |
| *F3´H* | Bra009312 | CTCCATCCACCAACACCACTC | TTTCCTTTCACATCCACGCC |
| *DFR* | Bra027457 | CTAAGGACCCCGAGAACGA | CCAGCAGACGAAGTAAACACA |
| *LDOX* | Bra013652 | CTATTACCCGAAATGCCCTCA | CTCCTTATTCACCAACCCACG |
|  | Bra019350 | GTTGACCTGATGGAGCGTG | AGCCGAGACCGACAGAGA |
| *BAN* | Bra021318 | GTTTCTATCAACAACCTTTCGG | ATCAATCTTATTCTCTTCCGC |
|  | Bra031403 | ATCAATCCAGCGATACAAGG | TTCGGTCATCACAAGTCCAG |
| *TT10* | Bra037510 | TCAATCGCAAAACACAGCC | CCCTCATTACCCTCTACCTCC |
| *TT12* | Bra003361 | CTAAGGCTGTTTGTGTGGGAG | GTAGAGGAACGTGAGGAGGAC |
| *TT19* | Bra008570 | CCAGTGGGCTGATGTTGA | AAACGGCGTCACATTCTT |
|  | Bra023602 | CTTGCTTTGTTTTTTGGAGAA | GTGCCTTGGTTGGAGTATTT |
| *AHA10* | Bra016610 | AGATTCATTTCTTGCCGTTCA | CTCCAGGACTATTACTGCTTCTTTC |
| *TT15* | Bra003021 | TTTCTTGACCCATTTGCTTC | CCTGACATTTCCTTCCCATC |
|  | Bra035004 | GACCAACTCCGACATACCATT | GCTGACATTTCCTTCCCATC |
|  | Bra038445 | TCATCGGACGAGGACTACATC | CCCACCACCAGACCTTTATC |
|  | Bra023594 | GGTCTTGCTTTACCTCGTGC | GACTCCAACCCTTCCGCTAT |
| *GAPDH* | GO0048316 | ATGATGATGTGAAAGCAGCG | TTTCAACTGGTGGCTGCTAC |
