# Supplementary material for: BrTTG1 regulates seed coat proanthocyanidin formation through a direct interaction with structural gene promoters of flavonoid pathway and glutathione S-transferases in Brassica rapa L
Source: Front Plant Sci. 2024 Apr 4;15:1372477. doi: 10.3389/fpls.2024.1372477 (PMC11024264; doi:10.3389/fpls.2024.1372477)
Supplement: Supplementary file 4 [file Table_2.docx]

Table S2: The promoter cloning primers used in this study.

| Primer name | Gene ID | Forwarding sequences5’-3’ | Reversed sequence5’-3’ |
| --- | --- | --- | --- |
| *proCHS* | Bra008792 | ACCTGGTGGGGAAATCATCACC | AGTATTACCAACTTGGTTTTAGTTACAAGAG |
|  | Bra006224 | GTCTCAAGCAACAGTTTCCTCAGAT | GTTTTACAAGAGTTTGATAGATGTGTGATC |
| *proDFR* | Bra027457 | CTGGGAAAGGACAGGGAGAAAAAAC | CTTTGTGTGTGAAAGATGGATTATGCTTTG |
| *proLDOX* | Bra013652 | GGGAAGACATTCGGAGATAAGACAA | CTTCTTGTACTTCTTGTAAAGCTAAAACAG |
|  | Bra019350 | CCTTCTTACTACGGAGACTGCATC | CTGATGAAAACAGAGTAAAGGTAAGGAAAG |
| *proBAN* | Bra021318 | ACAAGTCAGGAACGAAGCGTCC | CTGATTAAATTCTTAAGACACAGAAATTTTATATTTTGT |
|  | Bra031403 | CTCTCTCATCTTTGCCGTCTCAC | CTGATATATTATAAATTCTTAACGCACAGAAATG |
| *proTT12* | Bra003361 | TTTATTTTGTCTTATAGAAGACAAAATTTTAAATAGAACTAATT | GGTCCTCTTTTTTTTTTTTTTTTTTTTTCTCTTCTG |
| *proTT19* | Bra008570 | TTCCTCGTGCTGCTAACTGGAG | TCTATTACTTTGTAATTTTTTTTTTTGTATTTAATAGTATAAGA |
|  | Bra023602 | GCTTCATTGTCTCCTGGTAACTCT | TATAGTTTTTTGGTACAACTAACTTTGTAACAAC |
| *proAHA10* | Bra016610 | AGAGGACCAGAGAGATGGTTATGA | GCTTAAATCTTCAAAGACCACTGTCC |
