# Supplementary material for: BrTTG1 regulates seed coat proanthocyanidin formation through a direct interaction with structural gene promoters of flavonoid pathway and glutathione S-transferases in Brassica rapa L
Source: Front Plant Sci. 2024 Apr 4;15:1372477. doi: 10.3389/fpls.2024.1372477 (PMC11024264; doi:10.3389/fpls.2024.1372477)
Supplement: Supplementary file 5 [file Table_3.docx]

Table S3: All primers used for recombinant plasmid construction in the yeast one-hybrid assay.

| Primer name | Gene ID | Forwarding sequences5’-3’ | Reversed sequence5’-3’ |
| --- | --- | --- | --- |
| *proCHS*- pAbAi | Bra008792 | GAAAAGCTTGAATTCGAGCTCACCTGGTGGGGAAATCATCACC | TACAGAGCACATGCCTCGAGAGTATTACCAACTTGGTTTTAGTTACAAGAG |
|  | Bra006224 | GAAAAGCTTGAATTCGAGCTCGTCTCAAGCAACAGTTTCCTCAGAT | TACAGAGCACATGCCTCGAGGTTTTACAAGAGTTTGATAGATGTGTGATC |
| *proDFR*- pAbAi | Bra027457 | GAAAAGCTTGAATTCGAGCTCCTGGGAAAGGACAGGGAGAAAAAAC | TACAGAGCACATGCCTCGAGCTTTGTGTGTGAAAGATGGATTATGCTTTG |
| *proLDOX*- pAbAi | Bra013652 | GAAAAGCTTGAATTCGAGCTCGGGAAGACATTCGGAGATAAGACAA | TACAGAGCACATGCCTCGAGCTTCTTGTACTTCTTGTAAAGCTAAAACAG |
|  | Bra019350 | GAAAAGCTTGAATTCGAGCTCCCTTCTTACTACGGAGACTGCATC | TACAGAGCACATGCCTCGAGCTGATGAAAACAGAGTAAAGGTAAGGAAAG |
| *proBAN*- pAbAi | Bra021318 | GAAAAGCTTGAATTCGAGCTCACAAGTCAGGAACGAAGCGTCC | TACAGAGCACATGCCTCGAGCTGATTAAATTCTTAAGACACAGAAATTTTATATTTTGT |
|  | Bra031403 | GAAAAGCTTGAATTCGAGCTCCCGCAAACGCTAGCTGGAAC | TACAGAGCACATGCCTCGAGCTGATATATTATAAATTCTTAACGCACAGAAATG |
| *proTT12*- pAbAi | Bra003361 | GAAAAGCTTGAATTCGAGCTCTTTATTTTGTCTTATAGAAGACAAAATTTTAAATAGAACTAATT | TACAGAGCACATGCCTCGAGGGTCCTCTTTTTTTTTTTTTTTTTTTTTCTCTTCTG |
| *proTT19*- pAbAi | Bra008570 | GAAAAGCTTGAATTCGAGCTCTTCCTCGTGCTGCTAACTGGAG | TACAGAGCACATGCCTCGAGTCTATTACTTTGTAATTTTTTTTTTTGTATTTAATAGTATAAGA |
|  | Bra023602 | GAAAAGCTTGAATTCGAGCTCGCTTCATTGTCTCCTGGTAACTCT | TACAGAGCACATGCCTCGAGTATAGTTTTTTGGTACAACTAACTTTGTAACAAC |
| *proAHA10*- pAbAi | Bra016610 | GAAAAGCTTGAATTCGAGCTCGGTTGAAGCCTGCTCGTAGAAC | TACAGAGCACATGCCTCGAGGCTTAAATCTTCAAAGACCACTGTCC |
| pGADT7-*TTG1* |  | CCATGGAGGCCAGTGAATTCATGGACAACTCAGCTCCGGAC | AGCTCGAGCTCGATGGATCCTCAAACTCTAAGGAGCTGCATTTTGTTA |
