# Supplementary material for: BrTTG1 regulates seed coat proanthocyanidin formation through a direct interaction with structural gene promoters of flavonoid pathway and glutathione S-transferases in Brassica rapa L
Source: Front Plant Sci. 2024 Apr 4;15:1372477. doi: 10.3389/fpls.2024.1372477 (PMC11024264; doi:10.3389/fpls.2024.1372477)
Supplement: Supplementary file 6 [file Table_4.docx]

Table S4: All primers used for recombinant plasmid construction in the dual-luciferase assays.

| Primer name | Gene ID | Forwarding sequences5’-3’ | Reversed sequence5’-3’ |
| --- | --- | --- | --- |
| *proCHS*- LUC | Bra008792 | GGGCCCCCCCTCGAGGTCGACACCTGGTGGGGAAATCATCACC | CGCTCTAGAACTAGTGGATCCAGTATTACCAACTTGGTTTTAGTTACAAGAG |
| *proDFR*- LUC | Bra027457 | GGGCCCCCCCTCGAGGTCGACCTGGGAAAGGACAGGGAGAAAAAAC | CGCTCTAGAACTAGTGGATCCCTTTGTGTGTGAAAGATGGATTATGCTTTG |
| *proTT12*- LUC | Bra003361 | GGGCCCCCCCTCGAGGTCGACTTTATTTTGTCTTATAGAAGACAAAATTTTAAATAGAACTAATT | CGCTCTAGAACTAGTGGATCCGGTCCTCTTTTTTTTTTTTTTTTTTTTTCTCTTCTG |
| *proTT19*- LUC | Bra008570 | GGGCCCCCCCTCGAGGTCGACTTCCTCGTGCTGCTAACTGGAG | CGCTCTAGAACTAGTGGATCCTCTATTACTTTGTAATTTTTTTTTTTGTATTTAATAGTATAAGA |
|  | Bra023602 | GGGCCCCCCCTCGAGGTCGACGCTTCATTGTCTCCTGGTAACTCT | CGCTCTAGAACTAGTGGATCCTATAGTTTTTTGGTACAACTAACTTTGTAACAAC |
| *proAHA10*- LUC | Bra016610 | GGGCCCCCCCTCGAGGTCGACGGTTGAAGCCTGCTCGTAGAAC | CGCTCTAGAACTAGTGGATCCGCTTAAATCTTCAAAGACCACTGTCC |
| TTG1-62-SK |  | TTCAGCGTACCGAATTGGTACCATGGACAACTCAGC TCCGGAC | ATCCCCCGGGCTGCAGGAATTCAACTCTAAGGAGCT GCATTTTGTTAGC |
