# Supplementary material for: BrTTG1 regulates seed coat proanthocyanidin formation through a direct interaction with structural gene promoters of flavonoid pathway and glutathione S-transferases in Brassica rapa L
Source: Front Plant Sci. 2024 Apr 4;15:1372477. doi: 10.3389/fpls.2024.1372477 (PMC11024264; doi:10.3389/fpls.2024.1372477)
Supplement: Supplementary file 7 [file Table_5.docx]

Table S5: All primers used for recombinant plasmid construction in the promoters activity assays.

| Primer name | Gene ID | Primer sequence5’-3’ | | Size |
| --- | --- | --- | --- | --- |
| *proCHS* | Bra008792 | F | TTGGGCCCGGCGCGCCAAGCTTACCTGGTGGGGAAATCATCACC | 1222bp |
|  |  | F2 | TTGGGCCCGGCGCGCCAAGCTTGTAAAACAACAGTGACGGTAGTGAATAG | 691bp |
|  |  | F3 | TTGGGCCCGGCGCGCCAAGCTTGTGAGGGGTGTGAGAAGTAAAAGAA | 421bp |
|  |  | F4 | TTGGGCCCGGCGCGCCAAGCTTACACTCCCTTCTCTTTTTCTCCTTTG | 143bp |
|  |  | F5 | TTGGGCCCGGCGCGCCAAGCTTACACATAACATACTACATAAAGTCACGCA | 69bp |
|  |  | R | GAATTCCCGGGGATCCGTCGACAGTATTACCAACTTGGTTTTAGTTACAAGAG |  |
| *proDFR* | Bra027457 | F | TTGGGCCCGGCGCGCCAAGCTTCTGGGAAAGGACAGGGAGAAAAAAC | 1541bp |
|  |  | F2 | TTGGGCCCGGCGCGCCAAGCTTGCTGTGGTATGTCTGAGATGTTGG | 1099bp |
|  |  | F3 | TTGGGCCCGGCGCGCCAAGCTTAACTCTGTGAGGTGCTTTCTCTCAT | 902bp |
|  |  | F4 | TTGGGCCCGGCGCGCCAAGCTTAATTCACTGGAGCTGGTTAAGAAATGG | 581bp |
|  |  | F5 | TTGGGCCCGGCGCGCCAAGCTTGGCAATTCTTGTCGCTTTCTCACC | 319bp |
|  |  | F6 | TTGGGCCCGGCGCGCCAAGCTTCAAACAATCAAGTCCTTAGCCAACTAAC | 194bp |
|  |  | F7 | TTGGGCCCGGCGCGCCAAGCTTAAATGTTTTCTTATAGCACGAAGATTTCCACC | 86bp |
|  |  | R | GAATTCCCGGGGATCCGTCGACCTTTGTGTGTGAAAGATGGATTATGCTTTG |  |
| *proTT12* | Bra003361 | F | TTGGGCCCGGCGCGCCAAGCTTTTTATTTTGTCTTATAGAAGACAAAATTTTAAATAGAACTAATT | 1431bp |
|  |  | F2 | TTGGGCCCGGCGCGCCAAGCTTGTCACCCGACCTAATAGACAACTC | 956bp |
|  |  | F3 | TTGGGCCCGGCGCGCCAAGCTTGGTAAAAGGTAACATGTTAAATTGTGTTATGG | 691bp |
|  |  | F4 | TTGGGCCCGGCGCGCCAAGCTTGTTGAGACTAGCAGTTGGATCTATATTC | 392bp |
|  |  | F5 | TTGGGCCCGGCGCGCCAAGCTTAGTAGACACCAAAGTATCAGACCAAAC | 173bp |
|  |  | F6 | TTGGGCCCGGCGCGCCAAGCTTACCTTCAACATATAACCAAAACAAACATTCGTAATA | 97bp |
|  |  | R | GAATTCCCGGGGATCCGTCGACGGTCCTCTTTTTTTTTTTTTTTTTTTTTCTCTTCTG |  |
| *proTT19* | Bra008570 | F | TTGGGCCCGGCGCGCCAAGCTTTTCCTCGTGCTGCTAACTGGAG | 1235bp |
|  |  | F2 | TTGGGCCCGGCGCGCCAAGCTTCGCAGATTCCAGAGAGAGTTGAA | 1000bp |
|  |  | F3 | TTGGGCCCGGCGCGCCAAGCTTTGGAATGTTATAATCTTAGATTACCAAAACAAG | 758bp |
|  |  | F4 | TTGGGCCCGGCGCGCCAAGCTTTGATACATTGGCTTGGCATAGAGC | 603bp |
|  |  | F5 | TTGGGCCCGGCGCGCCAAGCTTCCATCGGCTCGACCAAACAC | 316bp |
|  |  | F6 | TTGGGCCCGGCGCGCCAAGCTTTAAGATCGATAAACACAAATTTAAAATAACACATATAAATTG | 98bp |
|  |  | R | GAATTCCCGGGGATCCGTCGACTCTATTACTTTGTAATTTTTTTTTTTGTATTTAATAGTATAAGA |  |
|  | Bra023602 | F | TTGGGCCCGGCGCGCCAAGCTTGCTTCATTGTCTCCTGGTAACTCT | 1116bp |
|  |  | F2 | TTGGGCCCGGCGCGCCAAGCTTGGATGCCTACTTCTGCTACACTG | 928bp |
|  |  | F3 | TTGGGCCCGGCGCGCCAAGCTTGCTAACCAAGGAGGAGGAATAGC | 786bp |
|  |  | F4 | TTGGGCCCGGCGCGCCAAGCTTGCCTACTTGGGATACACGTTAGG | 665bp |
|  |  | F5 | TTGGGCCCGGCGCGCCAAGCTTCATTGGGTTAAATGAAATTTCTATTATAGAATTTTTTAT | 360bp |
|  |  | F6 | TTGGGCCCGGCGCGCCAAGCTTGAGATTTGAATGATATCCACGGTTAGTG | 187bp |
|  |  | F7 | TTGGGCCCGGCGCGCCAAGCTTGAGTCTATTACACATCTACTCTCACTTC | 94bp |
|  |  | R | GAATTCCCGGGGATCCGTCGACTATAGTTTTTTGGTACAACTAACTTTGTAACAAC |  |
| *proAHA10* | Bra016610 | F | TTGGGCCCGGCGCGCCAAGCTTAGAGGACCAGAGAGATGGTTATGA | 1249bp |
|  |  | F2 | TTGGGCCCGGCGCGCCAAGCTTTGGGAACTCAGAGGTTGTATTGTG | 934bp |
|  |  | F3 | TTGGGCCCGGCGCGCCAAGCTTAGAGTGGAGTGACCGTTAGATCAA | 495bp |
|  |  | F4 | TTGGGCCCGGCGCGCCAAGCTTATTTTCCTGTTTCGCTTCTGCACC | 278bp |
|  |  | F5 | TTGGGCCCGGCGCGCCAAGCTTTAAATCAAACCCTGGAGCCTAGAG | 60bp |
|  |  | R | GAATTCCCGGGGATCCGTCGACGCTTAAATCTTCAAAGACCACTGTCC | 大小  Size |
