# Supplementary material for: BrTTG1 regulates seed coat proanthocyanidin formation through a direct interaction with structural gene promoters of flavonoid pathway and glutathione S-transferases in Brassica rapa L
Source: Front Plant Sci. 2024 Apr 4;15:1372477. doi: 10.3389/fpls.2024.1372477 (PMC11024264; doi:10.3389/fpls.2024.1372477)
Supplement: Supplementary file 8 [file Table_6.docx]

**proCHS-Bra008792**

ACCTGGTGGGGAAATCATCACCAAAATCTAATAAAAACCGAAAAGGCTAACAAAAAAAAGAAGATCATAGTATATACTAATATACAAGACGTAAATATATAGTACGTAACTTTAAAAATATTAAACAAACAAGAGTACGTAACCATACAAAACCTTAAAGACTAAATATAAGATAAAGACACATGCGTGTACGCAAGAAACATGCCTCAGGCCGACACACGCTCAAATACCATCGTTTTGGCCGTCCCTGGTTACGGCAGGCAGAGCTATATTTCGGTAAGCGCGAGCTTTTGGTTCGAATGATTATAACAAGCGCTCTGGAGTGCCTACTTACCATTGAGGAAACACACTCAGGTTCTCGTGACTTCTAGCTTATAATGGCCCACGTCCATAATATTGTCCATAACCCCATATGGTGTCAGAGTCAAGTTCAAGGGCCCACCTTCTATTATGGGTCGATGCTATTATCGTTTCCAACAAAAATATCTAAACATGAATACCTAATCATGTTGCTAATCATGTTTGTGGCCGGTAAAACAACAGTGACGGTAGTGAATAGTGAGTACTGAGTACTAAACAAAGGTGTCACAAGCATATATATACTTAATGACAGAAAAAAAAAAAAAAAGCAAAACAAATGAATCTTGCGTTGTTTGGAAAACGAAAAACGAGGCGTACCTAAATGTAAGACTATTTTTTTTTTTGCTAAACGATGTAAGACTAGTTATTTAATTAGTTAGTTATAGGATTAGAAGGAAACTGAAGAAGAAAATTTGGTTAACGATGGAGTTAAGTATGCACGTGAGGGGTGTGAGAAGTAAAAGAAGCTATACAACTAACCCCTAGAAACTGACGAGTAGCTGTACGTAAAATCTTTTGATTTATACATAGAATAAATAATAAATACGAACATAGAAATGAATACGACTTGTTGGTGCGAATATACGTAAATTCAATAAAACCAACCATTCAAATTTTTTTGGTAAGTAGCGGAATAAAAGGGAAGCAAGAAGAGCCACAGGAAAGCGAGCTAACACATAGACACGTTGTGTTTCATCTACCCGTCCATCCAACCTACCACACTCCCTTCTCTTTTTCTCCTTTGTCATCTTCTTATATAATCTACCACTCCCCGCATGTTTTCTAAATATATACACATAACATACTACATAAAGTCACGCATTGATCAACCTCTTGTAACTAAAACCAAGTTGGTAATACT

**proCHS-Bra006224**

GTCTCAAGCAACAGTTTCCTCAGATTACCAGTTTTCTCATGAAGTAACCTTAACCGACTGTATGAACTCATAAACTCACTCAACACTTGTGCATGTTTCGCTGAAACACTCTCCAGCTGAAGCTTCTCTGAGTTGTACTGCTCCATTAGGTCATCCAAATCGGGGAATGACCACTCAGCTTCTTCAACAGGTTGCTCATAGCATATCATCTCACGTTTCGCAACGGAACCATTAGAGACCGTATCTCTTCCCAAACCACTCGTTGCTTCTTCCTCCACACTACGCTTGGAAAGAGCACAATCACCATGTAGTTGCTCAATCACAACCGGATCAGACATAAGGATACCTAATAAACAACATGAGAGTACAAACAACAAGAAAGAGTCAAACTTTGTACATAACTGAATCAATCACCTTGGTTCACAGCAGCAGGAGCAGAATGTACATCAGAAACAGAAGAGTTATCCTCTGTATTCTCCGGTACACCAGCAGCAGCAGCCAGGAAGCTGCTCTCCACATCGCTCAAAACAAGATCAGCTTCCTCCCAGAAACCAATCTCATCAGCTGCATCACACCTCACCTCAACATCATCCACCCATTTGAAGAATCCACAAGCCCCAAAACCCTAAAAAAAACACTAACTTTCATAAAACCATAACACTCTAAGGCTTAAAAGTTATCAATTGATCAAATAAAAAAAAAACCTTTTTGACGCCACATTGCAAGTAAGCTCGTCCAGAATCAGCCACGACTCTCCGGCAAGGTCCAGAGCCGCAGGGGCAATCGGGGATGGTGAAGGCGGGGCGGAATCTGATATCTTCGTCGGTTGCTTTGTCACACCACTTGAAGAAAGTGCACTTCTGAGCCTGAGAGAGATTCAAATCGTTAAGTGAGAGAATTCAATTCGCGTTTGATTCGATGGAATCGTGAAAGGAGTACGAACAGCAGGGCATTTGTAGAATCTTCTTCCGGGATTCTCGTGAGTGTTCGAGAGCTTGGTTTCGCAGAGACCTCCGCCGCAAGGGCAGTGGATCGACGGTGGAGGAGGATCGTCGTCGGCGTTGGATTTGAGAGGGCAGTCGCTGATCCAGTGGCCGGTTCGACGGCAGCGGAAGCAGTTGCCGGTTTGCATCCCGTCGTCACGCACTCGTCGGCGACATCCAACCTCCGTCTCATTTCTCAATGATGTTGTTATGGGCCTTGAGCTTATGGTAATTGGTTTTTGAAGCCCAATGATTTGCTCGTTGATGTGGCCCATTAAGAGTTTAAGTGGGCCATATTAGATTATAAGATTTAAACCCATCAAATTAAAACTAAGCAGATTTTTATTTTCTTCTAAATTATAAACAAGAATTCAGATGTTACAACTTGTGGTTACAAATTCCTTAACCTAACTATCTAATCAACGTTGATTACCTAACTTTGTTAGATCTAAATCTAAGATGAGATTACACTAGCCAGAAGAGGCTAGCTACTAACACTCTATCTGTCCGATGATGGTTAAGTACATAGACAAGCGCCGAAGCTCAAAGGCAGCTAACTAAGTGGATTCAATTATTCAAGTATCATTATGATGGATGGGGAGAGAAACGAGAAGTGCTAACGTAATCTTTGTTAGTTAGTTAGTTGTAGGATTAGAAGGTAAATGAAGAAGGATAATTAGATTAGTAAGATGCAGTTAAGTATGCATGTGTAGGAAGTATGGTGAAGAAACTACGCACACCTAACCCCTCGTAGTTGACGTATCCTTTGATGTACACATCGAATAATATAATAAATACAAACATATAAATAAATGTCTTTTTGTTGTGTAAAACGTAAAATCAATCAAACCAAGTAACCAACCATTCAATTTTCTTGGTAAGTAACGAAATAAAAGGGAAGCAATAAAGAGAGCCACAGAAAAGAGACCTAACAGAGAAACACGTAATGTTCCATCTGCCCATCCATTCAACCTACCACACTCCCATCCCCCTTCTCCTTTGTCCTCTTGTTATATAATCTCACTCACCGTTTGCTTTCTAAATATATACACATAACTTGCATACTACACAAGATCACACATCTATCAAACTCTTGTAAAAC

**proDFR-Bra027457**

CTGGGAAAGGACAGGGAGAAAAAACAGTTCACACCGGGAAAACCTATCTCAAAATCATGACGGAAGGACCAAAAAAATTAAGAAGAGGAGTAAGCGAGTGAGGAGATAGACCAACCAAAATAAATTGAACAACACACATCAAGTGCTAAAGAAAATATTAAACCGGTCGGGCGAACGCATACAAAAGATGAAACAAATCCAATATGAACCAGAGAAAAGGGCCAGATTAGTGACAAGTATATTAAATGATGTGCCACATCCGACAATTCAAGTGTTTTTGGACAATATATATGTATATGGATAAACCTCTATTTATGAGATAGTTTTTGGCTGTGAGTTAAGTGCATTACTAATATGGTATTAGAGTTCATGGTGAGTCTATCATATAATTTTCCACATAAACAGTTTCATACCTATATAGCATGTATAAGTGGTTCATATTGCTGTGGTATGTCTGAGATGTTGGGCTTGGGCTGTTTCCACTGGACCGTTTTCTACCCACATTTCGAGAGGAGGCTATTAAATGATATCCCACATTGAATAATTCAAGTGTCCTTAGACAATATATATGTGTATGGGTAAACCTTCACTCATAAGTTAGTTTTTGGGTGTGAATTAGGCCTATCACTAATAAAATAAAACTCTGTGAGGTGCTTTCTCTCATCAAAAACACTAGACAAACCGGAAAATAACCTTAGAACCGACCATAGTAACAAACCATTGAGCAGATCTAATGTGTGATCTCTAGAAGTGCATGAAGGCCATCTACAACATTCGGTTAGACAAATCTAGAATCTCCAATCTTATAGAAGCATATACTGAGGGCCATATAGGAAATAAGAATTCTTCCACTCCGTTTATAAAACACCACCGGAAACATCGATCAAACCACTCACTATTGATACGGAAACGACAAATAAAGACAAAGAGGAAGATTTCAGCTCCCCTGATTGAAAAATTCACTGGAGCTGGTTAAGAAATGGAAGGAAGTCCCAAATGGAATAACGAAGGCTGAAGAAAGATAGAAAGTGTTTGTGTCATGTCATGATATTTTAAAATAGTTTTTTTATTAAATAAGATAGCATGTTACATTTTAAAGAGAATCATACTAGTAATTATCTTGTTAGGCCAAAAAAAAAAAATCAATGTGCAAAGAAAATCCCTAATCGCCAAATATATTATAATAATAAAAATTTTGAAAACAATGAAAAGAAAAGTAGATGGCAATTCTTGTCGCTTTCTCACCAACCAAGCAACTTCGAGCCAACGCACATTTCATCCGCCGGTACAGTACAGCTACATCCAAAAGTAATAAAATCAAATCACACTTACCAGTTTGCCAAGTACCAAACAATCAAGTCCTTAGCCAACTAACGTTCCACACGTGCTTTGCCGGTAGGTACTCACGTGACGGTAGCTTCTTAATAGCAATGTTCAGGCTGTGGTACGTCTATAAAATGTTTTCTTATAGCACGAAGATTTCCACCGAAGCTACATAGTAAACTCTTTCTCAAAGCATAATCCATCTTTCACACACAAAG

**proLDOX-Bra013652**

GGGAAGACATTCGGAGATAAGACAAGTGTGGATATGAATTCCTAGGCAGATCTCTGATATTTTTTAAAATATCCACGTCAGCATTAAAGATTCACCACCTTTTTTAATCAGAAAAAAAAATAGACAATTTTATGCTAGGGATTAGTAATAAACTTGTTTGTAAATTTTTTTCAATAAAAATATTGTAAATCATATATAAAAATATTTATTCACTTTTAGAGCTATAAATAGAGTAAGAATTACTATGAAATGAGAGTTCTTGGTTAAAATAAAAAAACAATGTGATATAATTTCTTTTTTCAAAAAAAAAGAAAAGAAAGATAATTAGTCTTGTTGAAACTGAATCCAGTCAAAATATTTTGTCAAAAATAAAATAAAATGTGGTTATAGGTGGACACGCGGAAACATGATTCAGGCACGTGCACACGGGCGCAGAAGAAACGCCACCAAATGTGATGGTGATTGGTATATATTCTACTGGGCCTCTTTGGATGGGCTTAATATCAGCACCCAAACCAGGACCCGGTAGGCGGTAACTTCTTTTCAGAATTAAGAAAGGATGTTTAGGGGTGCGCACTTCGGTTACTTTTTCGGTTCGGTTCGGTTTGGTTAGTTCGATTCTAGTATTTTTCTAACTGAAGTAAACCAAAATTAGTTTGATTCGATTTGTGTTCGATTCGGTTTATATTCGGTTTGGTTTATATTCGGTTCGTTTTTTTTTATCGGTTTAATTAGATTCTTTTTAGTTTAATTTTTTTAAGAGAAATTATGTTTTACAACATAATTATGTAAACATAAACTATGTGAACTAAATTCAATATAAAAACTGAAACAAAATACTTTAAAAAGTCACAAATATATATAAGAATTAAAACAAATGAAAACAAACTAAAATTAAAAAAAAAACCCAACATCATTAGTAATGTCTCTTATATCATTAAAATTAAAAAGCTTGCAATGAATGAAATATTTTAAATCAAATATTTTGATAATTACATATTAGGTTAGAAGAATATATGTTAATGAATAGAAATGGAAAATATGTGGTTTAGTATTAGAATTTTTAGTACATTGGTATTACTAATATTTTTAATTTAAATAATTATAAAAACATATCGGTGAACCATTTGGGTTAGGAAAATCTTTAACCGAATAGTTTCAAATAGACTTTGATTCGGTTTGAATCGGTTTCGGTTTGGTTGGCTCGGTTTGACTCGGTTCGGTTCGGTTTTTTTGCCCACCCCTATGTTTGACCGTTGGGTGTTAGGGGGATGGTGAAACTATCAGTCAACCAAACCAACATTTCCCGTTGACCATTAAGTGTGCACTCACTTAGTCACTTACCTCTTCTTCTTCACACATTCACTAACCACCGGTAGCTCTACACTTCTTAATCCGTTAGTTAATTTCTTTTTTATGAAGTAATACTTCTGCTATAAAAAGCCCTCTGCACATTACATTTATTTGCAATAAAAACCGAGACAGAGTAAGTAGTTATTTTGTATTGCATAACTCTGTTTTTCCCCTGTTTTAAAAAGCTTCTTTACTTACTCTGTTTTAGCTTTACAAGAAGTACAAGAAG

**proLDOX-Bra019350**

CCTTCTTACTACGGAGACTGCATCTTGGACCCTCACACACAATCTCACTACCATCGATCTATAGAACCAGAAAAATTCATAAAATAGAAGCTTAGAAAAATAATATATCTATCTTGAGTGAAGCATAAAGGGAATGGAACCTTTAGTTTGAGTTTGAGCTTATCAAACTCTTCATCATTAAGGATTGGATTACCAGAAACATAAGCCATTGAAGCTTCCAAGAACCTCTGTTCATCAGAACCTGCGTTGTTCATAAAAGCAATGACTTTCTGCCTAAGAAACCAAAACAAAAGATATGACTATATAGTTGATTACTGTACTTAGCATCACAACACTGCTTCCTTCCCACATCAACTCCTCTTTAAGGTTATCAAACTCCTCATTCGACATAGTCGCCTTGCCATCATAATAGAAAGACTACAAGAAAGAAAGAAACAATGCTCAAGCTCTTCATGAAGATTTGTTACTTATATATAAAAAAAAAAAGAGGATGGTTGATGGGTTTATGTAGCCGTTATACTTGCAAGGCTTGGAGAAACTCTTGTTCCATTTCACCAATTGATTTCTTTTCAGCCTTGTTGATGCTGCAGTACGGCATGATATTGCTATCAACGTCTTCTCCTCCACCTTGACCTTAAAGTAAGACCAAACGACTTAAAACTCATAAACTTAAAAGGTTCATTACAACTTTGAATGAGTTCGAAACAGAGAGATATGATGAAGACCTGATTGCTCTGTGGTGGCCTTTGCTGGCAACAAGAAGAACCTTTTTCTCAAGGAGATCGACCTTCCAGGGCTGAGCTGAGCCCACTGAGTCCTCGGAGATGGAGAAGAGAGAGAAGCGGTGGGTTTCCGAGACACGGCGGAGAAAAGACGGGGGCTTGTCAAACTGAACAACATCTTGCTACCCATTGTCACCGGGAGAAGAAAGAAGTTTTGTCAAGATGGAACCTATGGTTGAAGATGAGTGTTGAGTTACTGTGAGCTTCAGGGTTTTTTTTTTCTGATTATTTTAATTTATTATTATTTTTATTAAAATTTTAAATTTGGAGGGAAGAAGAGATAAGAGAGGTGTGGAGATGAATTCTTATGAGATCTCTGATATTTTTTTCCCTTCTCATCAGTGAGAAAGAGATCTTCCACGTCAGCATTAATATCCACCCTTAATAAATTATAACACACAGAATATACTAGCTTATAGACAAAATTTTATGCTAGGTTCTATAATAATACAATCCCATATTAATATTTATATTACTAATAATTTTTTTATAATAATTATGTATTTTCAGATCTAAGCAGATTAAGAAATGAATTAATAAATAAAGTTTTAAAGAGAATAATATCATATAGTTTTGTTTTTATTTTCAAAAAACATGTTAGAAGATAATTATTCTGGCTAATAATAAAACCAGTCAAAATGTTTTAGTAATTAACTGGTTTTAAAATTATACTGATGAAGCCATATATTGTCGACACGTGGAGATACGATTTAGGCACGTGCACACGGACGCAGAAGAAACGCCACCTGATGTGATGTTGGGGTTCAAATCGGTTCGGTTCGATTTTACCGAATTGAACACCTTTAAATCATCGTCGATAGTGATTGGTTTATTATATTGGGCCTATTTAAATGAGCTTTTATAGCCCATACCAAACAAGGACCCGGGAACTCCCCCGTTTTGACCGTTAGATCTTATAAGGATGGTGAAACTATCAGTCAAACCAAACTATCATCTTCCGTTGACCATGAAGTGTGACACTCACTTACTTACCCAACCTTCTTCACACAATCCACTAACAACCGGTAGCTCTACACTTCTTAATACAACAACTAATCCTTCTTTTTAATTAAAAATAAAATAAAATGAGATATTTTTTGCTATAAGAAAGCCTTTGCACATTTCATTTACTGGCAGCAAATCTAAAACAGAAAGTAAGTACTCTGTTTTTTCCCCTGTTCTAAGCTTCTTTCCTTACCTTTACTCTGTTTTCATCAG

**proBAN-Bra021318**

ACAAGTCAGGAACGAAGCGTCCGAGCGAAGGGACTTCAAGGGACACGAACTCGAAACGGGCGAAGAAGATAGGAGAAGATTCAAAGAAGCCTGCTTTCCAGAGACTATGGACTGAAGAAGACGAGATCGCTGTCTTGCAAGGTATGATCGATTTCAAGAACGATACAGGGAACTCTCCTTACGACGACACCAATGCTTACTACGATTACATCAAGAATTCTATTAGCTTTGAGGTTAGTAAGAACCAGTTCATGGATAAGCTTAGGAGCTTAAAGAAGAAGTATATGGGTAAAGAGAAGCCTTCTTTTACCAAACCTCATGATCACAAGTCTTATAGATTGTGCAATTACATCTGGGGACCTGATGGGATGGGTCTTGACACTGCTGTTAAGTCCAACGGTGTGTCCAAAAAGAAGACCAAGAAGCTTGCCGATGATGATGGTAAGAGAGTGTTGGTTCGTGCCGACGAGAAATCTGATTGGTTTGGGAACTCGTTTCTTGTTAGAGGGATTGCCGCTTTTGGTGTTGATGAGCATTATGTCAAACAGAGATGGAGCTTGGTCCCCGTTGAGACCAAGAAGAGAGTTGAAGAGAAGGTTAAGATGCTGCGGGCTAAGGAAATTGAGTTTCTGTTGCAGAAGGCTAAGATTTTGCATGAAGTTACCTCTATGATCGCTGAAGCATCCAAGAACAAGCCATTCGAGTGATAGATTCGATTGGGTTTCAGTGCCAATGTCTATGCTGTTATTTCAACTTAGCATTTACTTTCTCTTCTTTATGATCTACTTACTTTGTAATTTTCTGGTTTATTTTGATCGCTTTCTCTCATATTAAACCCTCTTTATTGGATTTGTTGCTTACATATACAAACACCATTAAAAAAAAAAAAAAAATTTACTGTCAAGCCTAAGGCAATTAGGTGAAATATGAATAGATCTTAGCCATAGAGTTTCAGTTTTTGGGATATTTCTAACGTAAAGAACTGTTCGGTATTATGTTTAATCAAATGGGTTTTTTCTGCAAAATTTGAAGTCAAAAGATCAAAACTGGTTAATGAAGAACTCTTTTGTTACAAAAATCTTGTGATAACTTAGTAAATGATAACACATCTGCAAGTCAATAAAAAGTTCCGAGGCTTCTTCTGATTCTTAGGTGAGAAAGAAGGTAAACAAGTTAGTTGTGTCAACGGTCACACGTGCTTACCCTCTAAAAAGCCTTTTGATCAACGGTTGTACCAAATGAGCAGAGAGTCTATAAGCTTTGCAACTTTTTCTTTTACTTCCTATATAAAAGGCTGAAGACGTTAACCTCTTAACAAACAAAATATAAAATTTCTGTGTCTTAAGAATTTAATCAG

**proBAN-Bra031403**

GCCGTCTCACGCACAAACCCTAAAAAGCGCTTCTCCTCGGCACTTTCAATGACGAAGAAACGCAACCCTTTAGAAAACCCACCAGCTGCTTCTTCCAGCGACGACGACGACGACGAAGCCGACTCTTCCGCTGGAGAAGAAGAAGAAGAAGTTAATGATTCTTCATCAGAGGAAGAAGAAGAAGAAGACCAACCCAATCCTCCTTCCTCCTCCGCCGTCACAATCGCCATCCCTGGTAAAACCACCTCAGCCTCCGATTCAGTTTCTGGATCCGAAACCGAGACCGATTCCGAATCCGATACCAAGAAGGACAAGTCTCCGCCGCCGGCAACCAAATCGGGAACGAAGCGGCCCAGCGAAGGGACTTCGAAGGAAGCGAACTCAAAGCGAGCGAAGATCGGCGAAGACTCGAAGAAGCCTGCTGCTTTTCAGAGACTGTGGACCGAGGACGACGAGATCGCTGTCTTACAAGGCATGATCGATTTCAAGAAGGATACGGGGAACTCTCCTTACGACGACACCAACGCTTACTACGATTACATCAAGAAATCAATTAGCTTTGAGGTTAGTAAGAACCAGTTCATGGATAAGCTTAGGAGTTTGAAGAAGAAGTATATGGGCAAAGAGAAGCCTTCTTTCACCAAGCCTCATGACCAGAAGTCTTATAGATTGTGTAAGTCTATATGGGGACCTGAGGGGATGGTTCTTGAGTCCAACGGCAAGAAGACCAAGAAGGTTGGCTCTGTGAAGCAGCAAGAGCTCTCTTTTGCTTCTTCTCCCAATGGTAAAACGGTTGATGATGGTGGTGACATGTATGATTGGTTTGAGAAGTCGTTTCTTGTGCGAGGGATTGCGGGTTTCGGTGTCGATGAGAGTTATGTGAAACAGAGATGGAGGTTGGTCCCGGTGGAGACTAAGAGGAAAGTTGAAGAGAAGGTTAAGATGTTGCAGGCCAAGGAGATTGAGTTTGTGTTGCAGAAGACTGAGATTTTGCATGAAGTCACCTCTTTGATTGCTGAAGCATCTAAGAACAAGCCATTAGAATGATTGATTTGATCTACGGTTTCAATGCCAATTGTCTATGCCTCTCTATTGTTTTTTCAACTTAGGATTTTTACTTTTTCTTCTTTTATGATCTACTTACTGTGTAATTTTTCTGGTTCATTTAGATGGCTTTCTCTCATATGAAACTCTCTTATATTGGATTTGTTGCTAATCTATAATAACACAATAAAAATAAGATTACTGTCAATTGTCAAGCCTAAGGCAGTTAGGTGACATATGAACTAGATCCTAGCTATGGTCGAACTGTTGGGTATTTTTATGAAATGTAGGTAAGTATTGGTTTGGTTCGGGTAATATGAACAAGGTTCTAGCAATTTGTGTTTAATCAAATGGGTTTGTTCTGAAAAAATTCAAGTAAAAAGATAAAAAATTTGGTTAAAGAAGACGAACTTACAAAAATCTTGTGATAACTTAGTTAAGTGATAACAAATCTGCAAGTCAATAAAAAGTTACGAGTCTTTCTTCTGATTCTTAGGTGAGAATTTAGGAAGAGGAAGAAGGTGAACAAGTTAGTTGAGTCACGTGCTTACCTTCTAAAAAGCCTTTTTGATCAATGGTTGTACCAAATGAGAAGAGAGACTAAAAGCTTTGCAAATTTTACTTTTACTTCCTATATAAAAGTCTCAAGACGTTAAACCTCATAACAAACAAATATACATTTCTGTGCGTTAAGAATTTATAATATATCAG

**proTT12-Bra003361**

TTTATTTTGTCTTATAGAAGACAAAATTTTAAATAGAACTAATTAATTTACATCTCATTTAACTATTTTATCCTCGTGTGTAATTCTTTGATAATTATATTAAAAATGAAATTGTGATATCCTAAATACAAATTTATAATTCATTATTTTCATGCATGACTATTGAGAAATTATACATGTATAAAGAAGATATATATAGTTAATACATTGATATTTTCATTAAACAATTAGCTACATGGATAAAGTATCAAAATGACATTTATCATTAAAATTATATACTTCATATTTAAGGGGGAAATTGGTATATTATACACTCGATAGCTAATGCAATATAATTTATTAAATTTTGATATAGTGGGAAAGTTTATTATGAAATTATTAGAAAATGTTGGCAAAAAAAGAAGAAGAAATTATTAGAAAATAAAACGCTTTTAATAATTGGTGGAAATAATGAGTAATCTTCTGAGTTTGGTGGGTCACCCGACCTAATAGACAACTCATTGATTTCATCTTACGTCATGGACAGCTCATCCAATGCGAATAATTTCTTTTATTTTTAGATTTATCGTGTATATTTACAGTTGGGCCTCGCGAGCAAAGCAGTAATGGGTTCATACGACGTTTGTGGGCCACATTTCTGAAAAGGGTTTCAGTTCCTTTTTTTAGTTCATTATTATTACTCGTATCTCTATTATTGGCAAGTGGTAGTACAACTATGGTAACATGTTAAAAAAAAGTTTGGTAAAAGGTAACATGTTAAATTGTGTTATGGAGAAACTAATTGTGTTCATAAAAAAAGGAGAAACTATATATTAATCACTCATAAACTTCATTAATTAAACTCAATCAGATACAAACGATCTATCCTTTTCATTAAACGAATATGTCCTTTTCGTTAGTTAAACTCAGTCCGATACAAAACAATATATCTTTTTCAGTAGTTAAACTTCATTAATCACTATCTTTTATCATTTTCTTGACAATTATCTACGTGTAAAACACTATTTCTTCACCCATTTAATTTAGAGGATGTACAAAGGTTGAGACTAGCAGTTGGATCTATATTCATAATCGTTGACGAACATTAGGTATCAACAACAACTTTTTTTTTCTTTTCATGCAAGTATAATTCGTTTTATACCTTGCCACATATTAATTCGTTCGATATGATTAGTTTCTCAATCTAGTATAAATGTTTTTTTTTTTTGCTTAAAATCTAGTATAAATGTTTAGGTGGTAACAAGAGAATGGTTGTTACAGTAGACACCAAAGTATCAGACCAAACAGTCATGAAACATCTAAACCGTCAATTATATTTTTCATCAACGACTCGAACCTTCAACATATAACCAAAACAAACATTCGTAATATAATTCACGAATTGTCTATAACTAACAGAAGAGAAAAAAAAAAAAAAAAAAAAAGAGGACC

**proTT19-Bra008570**

TTCCTCGTGCTGCTAACTGGAGCTTCACTCAACGATGAACACATCCAGGAGACAAAGACTGCCGACCACTACCTCTGCTACATTGTGAAGCCAGGCGTTTTCTTCATCGGTTCTATCATTTCGCTTTTCACTGTAGGCCTCGGGATTGTCTACTACTTATGTTTGAATTCGGTTAAGCAGAACGTTGCTGCTGCGACAACGACGCCGGCTAACCATGGGATAGCAATGGGACAGGCGCAGATTCCAGAGAGAGTTGAAGATCCTGTCTTTGTTCTTGAAGATACTTACATGAGAAGACAGTTCACTTAAAAGAATACAGAACATGGCTTGCTTGTGAAGCACGTTAGGTGTTTTAGGAGGATATTTTAGATAAAAAGGGTTGGTGCTTGTTTTGTTTTTATCTTTGTGGTTCTTGTGTGAAAGGAAGACTATTGAGAGACTTGGTAGTTGGATTAATGAAACGATGATTAGTGTTGTTGGAATGTTATAATCTTAGATTACCAAAACAAGGTCACTTGTTATCGTCTTTGAAGTGTATAGTTTCTCCCATTCTTTGTCTTTTACTTATTTATGTGAATCTAGTATGGTTGTTGGCAAGTGTTTGTCTCTTTTATAAAAGTTAAATGACAAGTTGATACATTGGCTTGGCATAGAGCGCTTAGTACATTGATTGGTAAAGCATTTGCCAAAAATTATGATCCAGTCAACAGTTATTAGAAAGACATTTAGAAGATGTAAATGCTCTTTATTCAATATCTAATATTGGTATAGTATTTGCATTGTAAATATATAAAAATTAAAAATAATTTATATAATTGATTTTTTATTTAACACCAAAGTTATGTTTATATTCAGAATCTAAATTTATATTTTTTTAAATAAAATTTACAATAAAATTTTATATTTATATTTAACCTACCCATCGGCTCGACCAAACACGGTTTGACAAGAACTTTAACACATACATTTGAGGTTCTTGAAACTTATAAAGCTTATAGAGAAATTTAAATGATACCCACCGTTAGTGGAACAACCACCACATGCTTTACCCAACCAAGCCACAACAACCAGCTCTTATAAACGTTTTAGTCTAATAAAATGCCAACACATCTACCCATACTTCTTCTCAATTATAAATAAGATCGATAAACACAAATTTAAAATAACACATATAAATTGATCTTTGCTTTTTCTTATACTATTAAATACAAAAAAAAAAATTACAAAGTAATAGA

**proTT19-Bra023602**

GCTTCATTGTCTCCTGGTAACTCTCACTCTTTAACTTTATTTTTTTTGCAGTAAATTCCCTAGTTATAATGTCTGCTTACAAATGATGTTCTTCATGTTACGTTTAGGTTCACTTTTGTGATAGCTTTCCTCGTGCTTCTATCTGGAGCTGCACTAAACGATGAACACGCCGAGGAGGCAGCGTTTCCGGATGCCTACTTCTGCTACACTGTCGGTACTGGCGTTTTCTCTACCGGTTCTGTGCTGTCGCTTGTCACTATTGCGCTCGGGATTGTTTACTATCTATGTTTGAATTCGAGTAACCAAAACGTTGGTGTCACAAGGACGGTGGCTAACCAAGGAGGAGGAATAGCAATGGGACAGCCTCAGATTCCAGAGAGAGGAGAAGATCCTGTCTTTGTTCATGAAGATACTTACATGAGAAGACAGTTCACTTAAATTCCAGAACAGGGCCTACTTGGGATACACGTTAGGTTTAGGATGATATATTTAGATTAAAAAGAAGGCTTTGTCTGATTTTTACCCCATTTTGTATCTTTCTGATTTTTTTTTTTGTATATCATCTCCAACTTATTTCTATATTTGTCTTTATAATAGTAGTTAGATGCCATGTTCGAAACTTTGTCGGACGGTCGGTCCGGACCTAGCGACTTAAGACTACATCATCCCATTTCTATTTTTTCCTCAAAAATATAGATTGATATTTTTCTTCTATATTTTTAGTATATATTTAGAAATTTCTATTTTTAAAAAACACATTGGGTTAAATGAAATTTCTATTATAGAATTTTTTATTTTAGAAAAAAAACTGAAAAATACATTGGAGATTGTCTGCTTACTTACCAGACTTGGTAGTTAGTAATATAACCACCGGTCCGATCAAATACTGTATTTGTTATCGGAATTTTTATTTTATTTAAAATTTTATAGAGATTTGAATGATATCCACGGTTAGTGGAACACCCACCACATGCTCTACCAACCAACCAGTCACAACAACAACCAGCTCTTATAAATGTCTCGAGTCTATTACACATCTACTCTCACTTCTTCTCATATATGTTATTAACATTAATAAAAACGTTGTTACAAAGTTAGTTGTACCAAAAAACTATA

**proAHA10-Bra016610**

AGAGGACCAGAGAGATGGTTATGAGAAAGATTGAGTTGAGATAGATGGTGAAGTTTCAGAACAGCGAGAGGGAGTTTTCCCTTTAGTCTTCTAAAGGGCAAATAGATTGCAGTGATGTGGCTACTCGAGGAATCATCGCATGTTATTCCTTCCCATGCGCAACAGTCAATGGATGGATTCCAATTCAGAGGAGAAACAGAAGAAGAAACGTTGCCGGAGAACCACAGGAGAGATTCTTGATCTTGATGGTTGCAGCCAGCTTCTGAAACTGTAAGGAAGAGGAAAACAACACATAGAAGGAGTAAGCGAAGCATGTGGGAACTCAGAGGTTGTATTGTGGTTGATGAAGCTCTCACAAGACCTTTGGCTTTGAATATCATGTCCTCATCAAACAAGAACAAGACAAGAAAAGTCGTTATAGAAGACAGAAAAAAACTTATTGGCAATACAAGTGTTGACTAAGGGGGAACAGTCACACAACAAAATAAGACAACTTGTCACACTAGCATGACCAAGGCAAAATAGTCAACATCTTCATAACTAATGATACATTTGTTTTTTTTTCTCGAGACTTTTGTGAATTATCTTATACCAATTGGTCCATTGTTAATCAAATTTCTTTTAGTCATTTTCTACCTAATGTTAGATTCAGATTAATATCTGATGACTTATTTGGGTTGTAATCTGACCAGCCCATATAAACAATCTAGGCCCAAAGAATGCGGTCGCAAGTCCCTTGTCAACCACACTTGTGAGAGTGGAGTGACCGTTAGATCAACCACACTTCTCTGTTCCCACTAACTAACTCTTCCTCATTCCCATCTGCTGTGTTCATAGACACTGACGTTTGATCTTATTCAATAATGCCAATACCAAATGCGACTTGCAGGAGAGAATCTCATCTTCCTCTTACTCCATACCAACTGGTCAACCATGGAAAGGTGGTTTCAATATTCACTAACCTTTTTAAAATTTTCCTGTTTCGCTTCTGCACCTCAAGTAGCATTCTCGATGACAAAGATATTAGTAGGTTCTTGTTATCATATAAGATGCTGTTAAGTCCTTTGTTTCATTATAATTAACTCGCATCAACCTTATTAAATAAGTAGCATAAATAAATAAATAAATTATAGGTTTATTCTTATGTTCATTGAAGAATCTATTGTTTCTCTTCATTAGTTTCATGGTTTAAATCAAACCCTGGAGCCTAGAGTTTTGAAGTTGGACAGTGGTCTTTGAAGATTTAAGC
